# Monolayer MoS_2_ Fabricated by In Situ Construction of Interlayer Electrostatic Repulsion Enables Ultrafast Ion Transport in Lithium-Ion Batteries

**DOI:** 10.1007/s40820-023-01042-4

**Published:** 2023-03-31

**Authors:** Meisheng Han, Yongbiao Mu, Jincong Guo, Lei Wei, Lin Zeng, Tianshou Zhao

**Affiliations:** Shenzhen Key Laboratory of Advanced Energy Storage, Department of Mechanical and Energy Engineering, SUSTech Energy Institute for Carbon Neutrality, Southern University of Science and Technology, Shenzhen, 518055 People’s Republic of China

**Keywords:** Monolayer MoS_2_, Interlayer electrostatic repulsion, Co atoms doping, Surface-capacitance effect, Fast-charging lithium-ion batteries

## Abstract

**Highlights:**

- In-situ construction of electrostatic repulsion between MoS_2_ interlayers is first proposed to successfully prepare Co-doped monolayer MoS_2_ under high vapor pressure.
- The doped Co atoms radically decrease bandgap and lithium ion diffusion energy barrier of monolayer MoS_2_ and can be transformed into ultrasmall Co nanoparticles (~2 nm) to induce strong surface-capacitance effect during conversion reaction.
- The Co doped monolayer MoS_2_ shows ultrafast ion transport capability along with ultrahigh capacity and outstanding cycling stability as lithium-ion-battery anodes.

**Abstract:**

High theoretical capacity and unique layered structures make MoS_2_ a promising lithium-ion battery anode material. However, the anisotropic ion transport in layered structures and the poor intrinsic conductivity of MoS_2_ lead to unacceptable ion transport capability. Here, we propose in-situ construction of interlayer electrostatic repulsion caused by Co^2^+ substituting Mo^4+^ between MoS_2_ layers, which can break the limitation of interlayer van der Waals forces to fabricate monolayer MoS_2_, thus establishing isotropic ion transport paths. Simultaneously, the doped Co atoms change the electronic structure of monolayer MoS_2_, thus improving its intrinsic conductivity. Importantly, the doped Co atoms can be converted into Co nanoparticles to create a space charge region to accelerate ion transport. Hence, the Co-doped monolayer MoS_2_ shows ultrafast lithium ion transport capability in half/full cells. This work presents a novel route for the preparation of monolayer MoS_2_ and demonstrates its potential for application in fast-charging lithium-ion batteries.
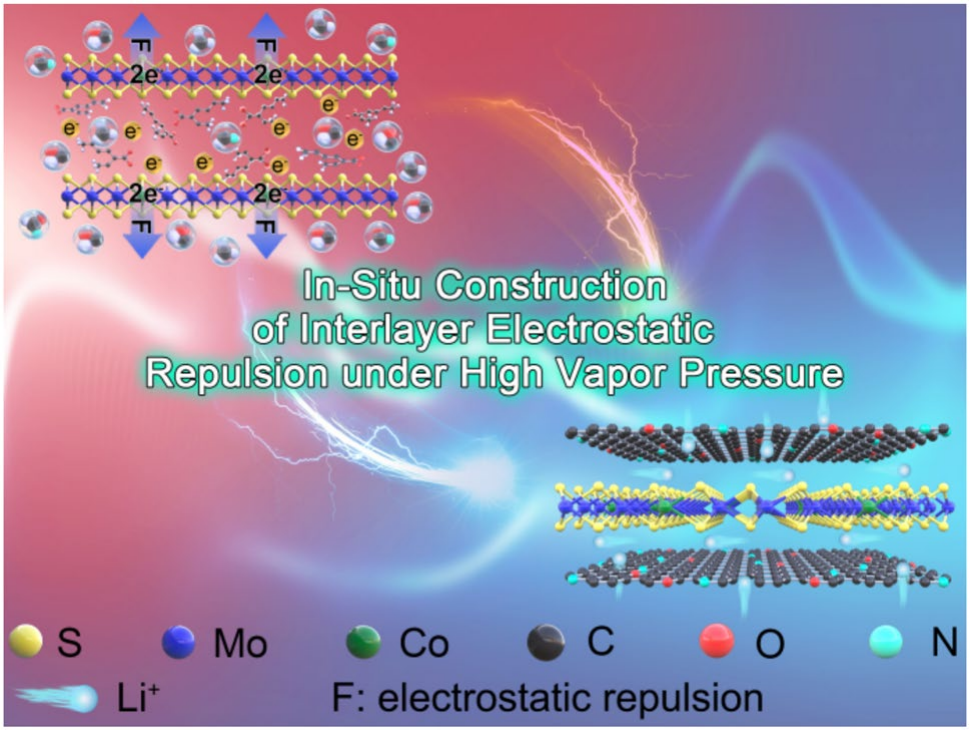

**Supplementary Information:**

The online version contains supplementary material available at 10.1007/s40820-023-01042-4.

## Introduction

Lithium-ion batteries (LIBs) have been extensively commercialized in portable electronics and electrical vehicles (EVs) [1]. Over the past decade, graphite has shown great success as the dominant anode material. However, its small interlayer distance (0.334 nm) and anisotropic ion transport channel have severely limited further advancement of the ion transport rate, which is unable to reach the requirements of fast-charging LIBs for EVs [2]. Moreover, its advancement in fast-charging LIBs has also been delayed owing to its low theoretical capacity [3]. To explore alternatives to graphite, high-performance anode materials such as metals [4], transition metal dichalcogenides (MoS_2_, etc.) [5–9], transition metal oxides [10], metal selenides (In_2_Se_3_, etc.) [11], SiO_x_ (x < 2) [12–14], and graphene [15] have been investigated, which are critical to break the fast-charging bottleneck of graphite LIBs.

MoS_2_ with interlayer spacing of 0.62 nm and lithium-ion storage capacity of 670 mAh g^−1^ looks very attractive [6, 7]. But the anisotropic ion transport channels caused by the layered nanostructure and poor electrical conductivity (EC) due to its inherent semiconducting properties cause unacceptable ion transport performance in fast-charging LIBs [9]. Based on these limitations, strategies such as designing MoS_2_ nanostructure [16, 17], enlarging MoS_2_ interlayer spacing [18], doping MoS_2_ [19], reducing MoS_2_ layers number [20], and fabricating composites of MoS_2_/carbon [6, 7, 9] have been proposed. Although these routes have greatly improved the lithium-ion transport capability of MoS_2_, the anisotropic ion transport features due to the native layered structure of MoS_2_ are not fundamentally changed, which inevitably leads to the same ion transport capability. The fabrication of monolayer MoS_2_ can be considered as the most effective route to circumvent the tricky problem due to the completely open ion transport pathway owing to the disappearance of layered structure [21]. The current preparation routes of monolayer MoS_2_ can be divided into two categories: (i) substrate growth, including chemical vapor deposition [21], epitaxy method [22],magnetron sputtering [23], etc.; (ii) bulk exfoliation, including scotch tape method [24], liquid ultrasonic method [25], chemically exfoliated method [26], etc. The first category is the synthesis of monolayer MoS_2_ membrane, not powder, which is inappropriate as LIB anode. The latter can achieve monolayer MoS_2_ powder but has low monolayer performance. Furthermore, the obtained monolayer MoS_2_ powder, which suffers from severe agglomeration, must be dispersed in solution and their poor EC is not improved, which is disadvantageous for application in fast-charging LIBs. In order to handle this tricky problem, the interoverlapped structure of monolayer MoS_2_ and monolayer carbon has been successfully constructed by solvothermal [27, 28] and with the help of CTAB [29], which can prevent monolayer MoS_2_ agglomeration and improve its EC. However, such interoverlapping structure reintroduces anisotropic ion transport features between monolayer MoS_2_ and monolayer carbon, which is unfavorable for the full reflection of intrinsic ion transport capability of monolayer MoS_2_. With the aim of solving this problem, monolayer MoS_2_/carbon composites, in which monolayer MoS_2_ is uniformly dispersed into carbon matrix, have been synthesized by electrospinning [30], dual-template route [31], and emulsion-templated solvothermal route [32]. This completely eliminates anisotropic ion transport features, thus obtaining high capacity (1267 mAh g^−1^ at 0.1 A g^−1^) and good rate capability (60.6% capacity retention at 10 A g^−1^ relative to the capacity obtained at 0.1 A g^−1^). Although the electrochemical performances of MoS_2_ have been improved in these researches, some problems are still waiting to be solved. Generally, under the same areal capacity, higher specific capacity makes active materials with less mass loading, thus resulting in thinner electrodes, which certainly increases the ion transport rate throughout the electrode. However, the addition of low-capacity carbon materials inevitably reduces the capacity of the composites, which has an adverse effect on rate performances. The introduction of active materials with high capacity has been the key to solving this problem. Additionally, the intrinsic semiconductor properties of monolayer MoS_2_ have not been fundamentally changed, which certainly decreases ion transport capability in basal plane of monolayer MoS_2_. For these problems, it is vital and urgent to seek a new preparative technology of monolayer MoS_2_ to spur monolayer MoS_2_ application in fast-charging LIBs.

Herein, we report a facile route that Co^2+^ in situ substitution of Mo^4+^ induces negative charges in MoS_2_ to in situ construction of electrostatic repulsion in the interlayer of MoS_2_ under high-pressure vapor phase, along with vast gaseous groups insertion, can effectively separate MoS_2_ layers, thus obtaining monolayer MoS_2_. Meanwhile, the gaseous groups can be transformed into N,O codoped carbon substrate to effectively suppress agglomeration and re-stacking of Co-doped monolayer MoS_2_, thus obtaining the unique nanostructure that Co-doped monolayer MoS_2_ uniformly dispersed into N, O codoped carbon matrix (CoMoS_2_/C). As LIB anode, the CoMoS_2_/C has the following advantages: i) monolayer MoS_2_ with fully exposed basal planes has an open Li^+^ transport path owing to the disappearance of anisotropic ion transport path in layered structure, thus ensuring ultrafast lithium-ion transport capability; ii) a maximized contact area of monolayer MoS_2_ and C can sufficiently improve the EC of MoS_2_, thus accelerating the charge transfer rate; iii) Co doping fundamentally changes the electronic structure of monolayer MoS_2_ to enhance its intrinsic EC, thereby remarkably accelerating the charge transport; iv) Co-doped monolayer MoS_2_ can be transformed into small superparamagnetic Co and Mo nanoparticles (~ 2 nm) during the conversion reaction, which can create a space charge region to accelerate charge transfer. The i-iv superiorities enable CoMoS_2_/C with ultrahigh capacity (1512.9 mAh g^−1^ at 0.1 A g^−1^) and ultrafast ion transport capability (1063.3 mAh g^−1^ at 20 A g^−1^) in half cells. Moreover, an energy density of 136.2 Wh kg^−1^ is acquired at 4 C in full cells with 76.6% retention corresponding to 0.1 C. Our work indicates that the in situ construction of electrostatic repulsion in the interlayer is a very effective route for the fabrication of monolayer MoS_2_ and reveals the application potential of Co-doped monolayer MoS_2_ in fast-charging LIBs.

## Experimental Section

### Samples Synthesis

The samples were fabricated using mixed precursor of Cobalt bis (2-ethylhexanoate), (NH_4_)_2_MoS_4_, and N,N-dimethylformamide (DMF) in a fixed total mass of 1.6 g with different mass ratio of 0:1:0, 0:1:3, 3:4:9, 5:4:7, and 7:4:5 to synthesize MoS_2_, MoS_2_/C, CoMoS_2_/C-I, CoMoS_2_/C-II, CoMoS_2_/C-III, respectively, in a self-made device that can be sealed. The devices were heated to 520 °C for 20 min in a tube furnace with an Ar flow and then experienced natural cooling.

### Characterizations

The obtained materials were tested by scanning electron microscopy (SEM, FEI Quanta 450 FEG), thermogravimetric analysis (TGA, Pyris I, PerkinElmer), elemental analyzer (EA, PerkinElmer 2400 Series II), X-ray diffraction (XRD, D/max-2500/PC, Rigaku), X-ray photoelectron spectroscopy (XPS, Thermo Fisher ESCALAB Xi^+^), Raman spectroscopy (Horiba LabRam HR Evolution), electron spin resonance spectroscopy (ESR, Renishaw RM-1000), photoluminescence spectra (PL, Bruker EMXplus), Brunner–Emmet–Teller method (ASAP 2020, HD88), transmission electron microscopy (TEM, Hitachi HT7700) with an energy-dispersive spectroscopy (EDS), and physical property measurement system (PPMS-14L, Quantum Design).

### Electrochemical Measurements

#### Half Cells

The working electrodes were made by spreading slurries of MoS_2_-based composites, acetylene black (AB), and polyvinylidene fluoride (PVDF) with a mass ratio of 8:1:1 on Cu foil and dried at 100 °C under vacuum with a time of 12 h. Working electrode, counter/reference electrode (lithium foil), separator (Celgard 2400), and electrolyte were assembled in 2032 coin-type cells, with the active mass of 1.5 mg cm^−2^. The lithium-ion storage performances were tested by Land CT2001A battery system at 0.1–20 A g^−1^ with a voltage window of 0.01 to 3 V (*vs*. Li/Li^+^). A CHI 760D electrochemical workstation was used to test cyclic voltammetry (CV) at 0.1–1 mV s^−1^ with the same voltage window and electrochemical impedance spectroscopy (EIS) from 10^5^ to 10^−2^ Hz with 5 mV amplitude.

#### Full Cells

Coin-type full cells were assembled with LiFePO_4_ cathode made by spreading the slurries of 95.0 wt% LiFePO_4_, 2.5 wt% PVDF, and 2.5 wt% AB on Al foil and CoMoS_2_/C-II anode with a N/P ratio of ~ 1.06, in which the active mass loading of anodes and cathodes are 2.0 and 16.4 mg cm^−2^, respectively. Other components are the same as those of half cells. Before packaging, the anode was pre-cycled (3 cycles at 0.1 A g^−1^) to ameliorate its first Coulombic efficiency (CE). The performances were tested at 0.1–4 C (1 C = 170 mA g^−1^) with a voltage window of 1.0 to 4.0 V at 30 °C.

### Simulation Method

Density functional theory (DFT) is executed in view of Vienna Ab-initio Simulation Package. Interactions between e^−^ and Li^+^ are detailed by Projected Augmented-Wave potentials, meanwhile exchange–correlation interactions are calculated by executing Perdew–Burke–Ernzerhof pseudopotentials of Generalized Gradient Approximation. DFT-D3 method is applied to determine the van der Waals (vdW) interaction. Plane-wave energy cutoff and convergence threshold are considered as 450 eV, and 1.0 × 10^−5^ eV in energy and 0.02 eV per Angstrom in force, respectively. Brillouin zone is sampled with 4 × 4 × 1 k-points. A vacuum space of 3 nm is inserted in z direction to prevent interactions between periodic images. Migration energy barrier of Li^+^ is calculated by executing climbing image nudged elastic band (CI-NEB) method, and the force is approximate to 0.3 eV nm^−1^.

## Results and Discussion

### Materials Analysis and Formation Mechanism

**Fig. 1:**
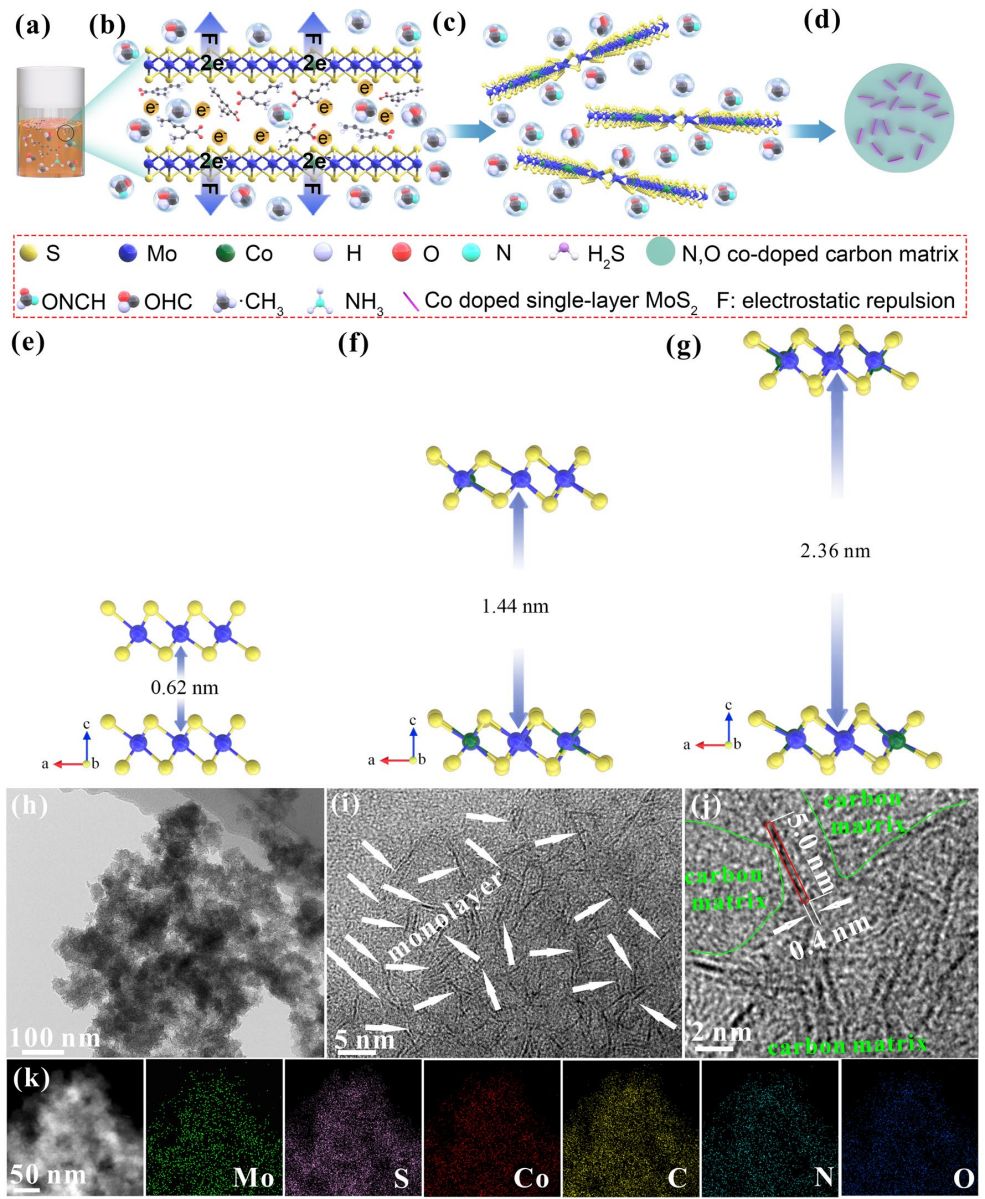
**a–d** Schematic diagram of formation process of monolayer MoS_2_. DFT results of **e** pure MoS_2_, **f** Co-doped MoS_2_ with a Co/Mo atomic ratio of 1/5, and **g** Co-doped MoS_2_ with a Co/Mo atomic ratio of 1/2. **h** TEM image, **i**, **j** HRTEM images, and **k** HAADF and its EDS mapping images of CoMoS_2_/C-II

Figure S1 displays the morphology of the obtained samples, where pure MoS_2_ exhibits bulk morphology with size of over ten microns (Fig. S1a, b). Clearly, MoS_2_/C exhibits spherical shape with sub-micron size and maintains the morphology of nanosheets (Fig. S1c). This indicates that MoS_2_ is not completely embedded in carbonaceous materials due to the low mass of carbon materials arising from DMF [9]. Differently, the CoMoS_2_/C samples show no obvious flaky morphology (Fig. S1d–f) due to the increase in mass of carbon materials (confirmed by TGA curves below) arising from cobalt bis (2-ethylhexanoate) and DMF, which fully wraps the MoS_2_ nanosheets. Clearly, the CoMoS_2_/C-III also exhibits the appearance of a peculiar bulk with a size of ~ 200 nm marked with yellow circles (Fig. S1f), which is attributed to the formation of Co_3_S_4_ (confirmed below) due to the excessive addition of C_16_H_30_CoO_4_. The microstructures are observed via TEM, in which the pure MoS_2_ is multilayered (~ 15 layers) with a d-spacing of 0.62 nm (Fig. S2). After bringing in DMF, few-layered MoS_2_ (~ 5 layers) with an enlarged *d*-spacing of ~ 0.96 nm and distributed into the carbon substrate is obtained in MoS_2_/C (Fig. S3). After drawing into a small amount of C_16_H_30_CoO_4_, few-layered MoS_2_ (~ 5 layers) with a larger *d*-spacing (1.16 nm) and distributed into the carbon substrate are attained in CoMoS_2_/C-I (Fig. S4). As the amount of C_16_H_30_CoO_4_ continues to increase, the intrinsic layered structure of MoS_2_ completely disappears, so monolayer MoS_2_ is formed in CoMoS_2_/C-II (Fig. 1). After bringing in excess C_16_H_30_CoO_4_, the Co_3_S_4_ is formed besides monolayer MoS_2_ (Fig. S5). The results indicate that monolayer MoS_2_ can be successfully fabricated by varying the amount of organic ionic liquid (C_16_H_30_CoO_4_ containing one positively charged Co^2+^ and two negatively charged (CH_3_)_2_C(CH_2_)_4_COOH^−^) in the solution of (NH_4_)_2_MoS_4_ and DMF. It should be noted that the source mass of Co and DMF is simultaneously changed, which is mainly from the perspective of ensuring the experimental safety and the success of monolayer MoS_2_ synthesis. Since the maximum capacity of our reaction device is 5 mL, according to our experience in vapor-phase high-pressure reaction, the total volume of added liquid cannot exceed 30% of the total capacity. Otherwise, the pressure of the vapor phase generated by liquid pyrolysis would exceed the maximum resistance pressure of the device, which causes safety hazards. In addition, the pressure of the vapor phase generated in the reaction is also important for the synthesis of monolayer MoS_2_. Although the mass ratio of cobalt bis (2-ethylhexanoate)/(NH_4_)_2_MoS_4_/DMF remains unchanged, the decrease in total mass leads to a low vapor pressure that is insufficient to form monolayer MoS_2_ (Fig. S6). Therefore, appropriate total precursor mass and liquid volume are very important for experimental safety and synthesis of monolayer MoS_2_. Based on the above discussion, the samples are synthesized with the same total mass of the precursors, and with the increase in the mass of Co source, and the mass of DMF decreases.

Accordingly, the formation mechanism of monolayer MoS_2_ should be explored, as shown in Fig. 1a–d. Specifically, in a sealed vessel, with the increase in temperature, the pyrolysis of (NH_4_)_2_MoS_4_, DMF, and C_16_H_30_CoO_4_ starts (Fig. 1a), during which (NH_4_)_2_MoS_4_ can be decomposed into NH_3_, H_2_S, and MoS_2_ [9]; DMF can be decomposed into OHCN, OHC·, ·CH_3_, etc. [9]; the negatively charged carbon chain in C_16_H_30_CoO_4_ can be decomposed into OHC·, ·CH_3_, etc. The production of vast gases and gaseous groups leads to high vapor pressure in the sealed vessel [33], which can drive the produced gaseous groups and Co^2+^ into the nucleating and growing MoS_2_ interlayers (Fig. 1b). The in situ replacement of a Mo^4+^ by a Co^2+^ leaves two negative charges on the MoS_2_ layers, which creates an electrostatic repulsion force between MoS_2_ base plane and the two negatively charged groups initially coordinated to the Co^2+^, thus increasing the interlayer spacing (Fig. 1b). By increasing the amount of Co^2+^ doping, the electrostatic repulsive force can be further increased, and after reaching a threshold value that makes the interlayer van der Waals force disappear completely, it brings about the formation of MoS_2_ monolayer (Fig. 1c). Moreover, the gaseous groups produced by the pyrolysis of precursors insert the MoS_2_ interlayer, which facilitates the effective separation of monolayer MoS_2_. Meanwhile, the gaseous groups are filled around the monolayer MoS_2_, which effectively prevents their agglomeration and re-stacking. With the further increase in temperature, higher vapor-phase pressure can be generated, which can induce the transformation of carbon-containing gaseous groups to solid carbon materials (Fig. 1d).

To confirm the mechanism by which the electrostatic repulsion force can be in situ built after introducing Co^2+^ into MoS_2_ lattice, the DFT calculations are performed. The equilibrium interlayer spacing of adjacent MoS_2_ with different additional charge concentrations caused by Co doping is investigated and acted as the basis for the construction of MoS_2_ model. Specifically, when a Co^2+^ replaces a Mo^4+^ of the MoS_2_ layers in the doping form, the MoS_2_ layers carry two negative charges. Meanwhile, the donor (cobalt bis (2-ethylhexanoate)) of one Co^2+^ provides two negative charges in the interlayer and two loads on MoS_2_ layers, leaving four charges for the model. The electrostatic repulsions are triggered via the accumulated negative charges to augment *d*-spacing of MoS_2_. MoS_2_ retains an interlayer spacing of 0.62 nm (Fig. 1e). Co-doped MoS_2_ model with the Co/Mo atomic ratio of 1/5 is constructed and the corresponding negative charges are introduced to the model (Fig. 1f). It is worth noting that the MoS_2_ interlayer distance calculated from the model should be lower than the experimental result in CoMoS_2_/C-II. This is because the latter is motivated by a combination of gaseous group insertion and interlayer electrostatic repulsion instead of only electrostatic repulsion for the former. However, the calculated interlayer distance (1.44 nm) is slightly higher than 1.16 nm of CoMoS_2_/C-II under a similar amount of Co doping (based on XPS results below). This may be due to the volume shrinkage arising from the conversion from gas to solid phase under high vapor-phase pressure and the incomplete insertion of negatively charged gaseous groups in the interlayer. Continuing to increase the amount of Co atoms and negative charges in the model (1/2, atomic ratio of Co/Mo), the interlayer distance is further increased to 2.36 nm (Fig. 1g), which signals the formation of monolayer MoS_2_ [34]. The DFT results fully identify the validity of the interlayer electrostatic repulsion in enhancing the interlayer spacing of MoS_2_, even in fabrication of monolayer MoS_2_. As observed in the TEM images (Fig. 1h–j), Co-doped monolayer MoS_2_ (Fig. 1i, pointed by white arrows) with a linear size of ~ 5.0 nm, breadth size of 0.4 nm, dispersed in the carbon matrix (circled by green line) (Fig. 1j) is fabricated. High-angle annular dark field (HAADF) and corresponding EDS elemental mapping images (Fig. 1k) display that six elements are uniformly released from the composite, signifying the homogeneous dispersion of the Co-doped monolayer MoS_2_ into N,O codoped carbon substrate. The carbon materials are diffuse, indicating that they are amorphous, as confirmed by the XRD pattern below.

**Fig. 2:**
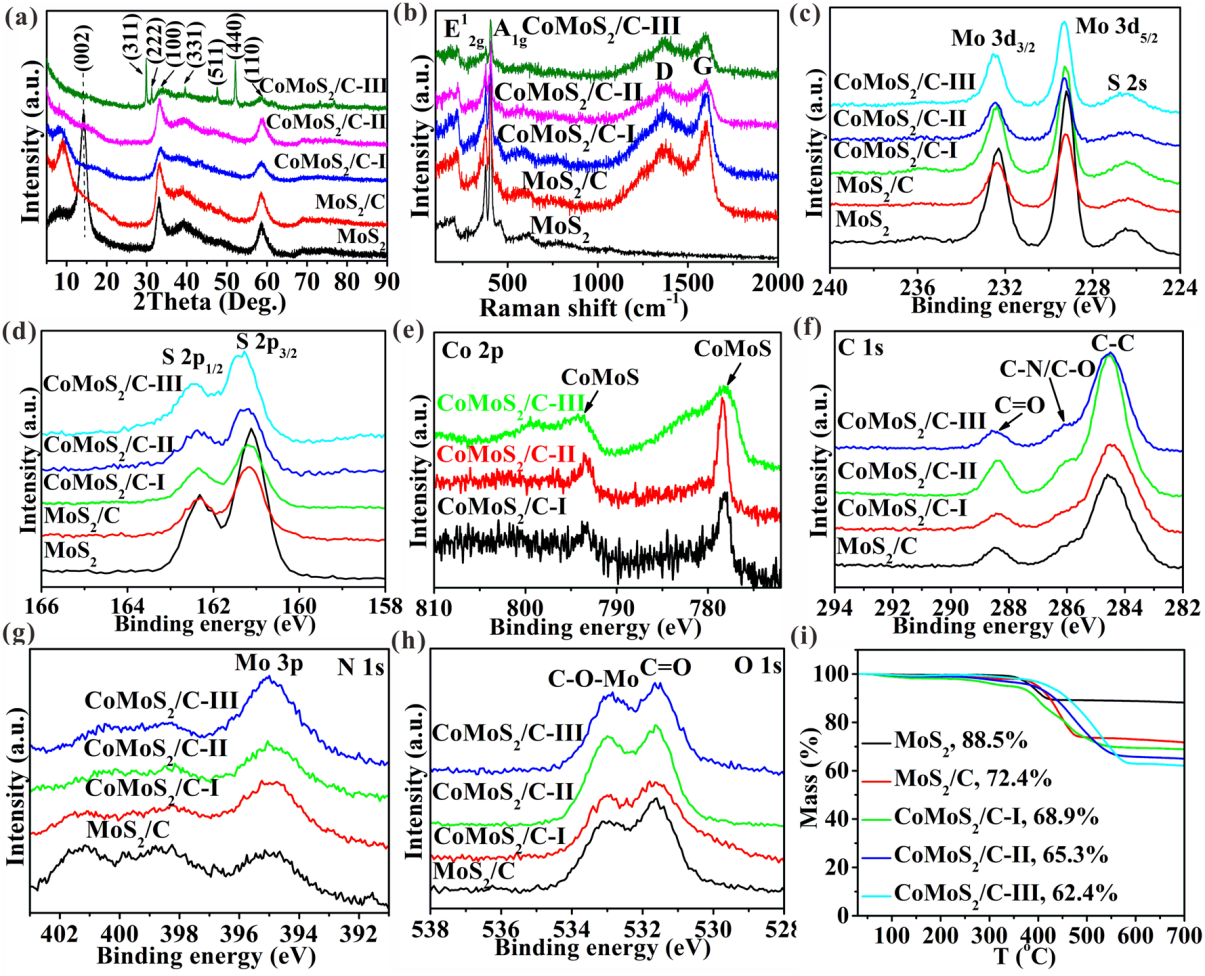
Sample analysis. **a** XRD, **b** Raman, XPS spectra of **c** Mo 3*d*, **d** S 2*p*, **e** Co 2*p*, **f** C 1*s*, **g** N 1*s*, **h** O 1*s*, and **i** TGA

Figure 2a displays XRD patterns in which the diffraction peaks for pure MoS_2_ belong well to 2H-MoS_2_ (JCPDS:37–1492), where the diffraction peaks at ~ 14.2, 33.5, and 58.5° correspond to (002), (100), and (110) of MoS_2_ crystal planes, respectively. Clearly, the diffraction peak of the (002) crystal plane for MoS_2_/C shifts to 9.0°, which corresponds to d-spacings of ~ 0.96 nm calculated by Bragg’s Law (2*d* sin*θ* = *nλ*), further confirming the enlargement of the interlayer spacing. Obviously, compared with the MoS_2_/C, the diffraction peak of the (002) plane of the CoMoS_2_/C-I shifts to 7.8°. This indicates a larger interlayer spacing (1.16 nm), confirming the role of Co doping enhancement in increasing the d-spacings. Interestingly, the (002) plane of CoMoS_2_/C-II and CoMoS_2_/C-III completely disappears, which belongs to the feature of monolayer MoS_2_ [30, 32] and further confirms the formation of monolayer MoS_2_. Besides, the CoMoS_2_/C-III shows the peaks of Co_3_S_4_ (JCPDS No. 02-1338) to further testify the formation of Co_3_S_4_. Clearly, no obvious diffraction peaks of carbon materials appear in the XRD pattern, which indicates that the carbon materials are amorphous. Figure 2b exhibits Raman spectroscopy, in which all the obtained samples show *E*^1^_2g_ (~ 378 cm^−1^) and *A*_1g_ (~ 401 cm^−1^) Raman peaks of MoS_2_ [9]. This further confirms the presence of MoS_2_. Besides the pure MoS_2_, all other samples show the peaks of disordered carbon (~ 1367 cm^−1^, D-band) and ordered graphitic carbon (~ 1600 cm^−1^, G-band) of carbon materials [14], further confirming the formation of carbon in these samples. The frequency differences between *E*^1^_2g_ and *A*_1g_ vibrations are related to the layer number of MoS_2_, which are 27.2, 24.6, 23.7, 20.6, and 20.6 cm^−1^ for pure MoS_2_, MoS_2_/C, CoMoS_2_/C-I, CoMoS_2_/C-II, and CoMoS_2_/C-III, respectively (Fig. S7). In previous reports [35–38], the frequency differences were > 27.0, 23.4–26.7, ~ 22.0, and 20.3–20.7 cm^−1^ corresponding to the multilayered, few-layered (3–6 layers), bilayered, and monolayer MoS_2_, respectively. The results further demonstrate the presence of multilayered MoS_2_ in pure MoS_2_, few-layered MoS_2_ in MoS_2_/C and CoMoS_2_/C-I, and monolayer MoS_2_ in CoMoS_2_/C-II and CoMoS_2_/C-III. Moreover, the disorder degree of graphitic structure can be validated by the intensity ratios of D and G peaks (*I*_D_/*I*_G_) [14, 39, 40], which are 0.83, 0.91, 0.96, and 0.99 for MoS_2_/C, CoMoS_2_/C-I, CoMoS_2_/C-II, and CoMoS_2_/C-III, respectively. Noticeably, the I_D_/I_G_ ratio shows an upward trend with increasing C_16_H_30_CoO_4_ amount. This may be because the carbon-containing gaseous groups arising from the pyrolysis of C_16_H_30_CoO_4_ have a greater tendency to transform into disordered carbon under high vapor pressure, thus increasing the ratio I_D_/I_G_. So high *I*_D_/*I*_G_ values suggest the presence of extensive defects in carbon materials. Moreover, the defects in MoS_2_ can be evidenced by the ESR (Fig. S8a) and PL spectra (Fig. S8b). Compared with pure MoS_2_, the ESR spectra of MoS_2_/C and CoMoS_2_/C show a stronger characteristic peak intensity (*g* = 2.003). This confirms the presence of abundant non-intrinsic defects in MoS_2_ [41]. Moreover, the PL spectra show that the MoS_2_/C and CoMoS_2_/C have a lower emission peak intensity at ~ 625.0 nm compared to pristine MoS_2_, which is related to bandgap transition of MoS_2_. This indicates the effective prevention of free electron-hole pairs due to the presence of defects [42]. Especially, a stronger peak intensity in ESR and a lower peak intensity in PL of the CoMoS_2_/C samples than MoS_2_/C are owing to Co doping. The above results suggest that compared with pure MoS_2_, vast defects exit in MoS_2_/C and CoMoS_2_/C samples, which is advantageous for charge transfer and storage.

From the XPS survey spectra (Fig. S9), all samples show their proper element signals besides MoS_2_, in which the presence of C and O should be due to adventitious impurities [9]. Besides, by fitting the XPS survey peaks, the atomic percentages of each element can be calculated, as shown in Table S1. The corresponding Co doping amount in the MoS_2_ and the amount of N,O doping in carbon materials are also calculated and are shown in Tables S2 and S3, respectively. Apparently, the amount of Co doping in MoS_2_ increases (5.80 at%, CoMoS_2_/C-I; 11.52 at%, CoMoS_2_/C-II; 21.69 at%, CoMoS_2_/C-III) (Table S2) due to the increase in the addition amount of C_16_H_30_CoO_4_, while the amount of N,O doping in C decreases (Table S3), which is attributed to the decrease in the addition amount of DMF. The spectra of Mo 3*d* (Fig. 2c) and S 2*p* (Fig. 2d) reveal typical 2H-MoS_2_ peaks at ~ 232.5 (Mo 3*d*_3/2_), ~ 229.3 (Mo 3*d*_5/2_), ~ 162.3 (S 2*p*_1/2_) ~ 161.2 (S 2*p*_3/2_) eV [9]. The CoMoS_2_/C samples show clear Co 2*p* XPS spectra (Fig. 2e) at ~ 793.6 and ~ 778.5 eV which are attributed to the presence of CoMoS [43], indicating that Co replaces Mo in the doped form. Differently, the CoMoS_2_/C-III shows two peaks at 782.3 and 789.6 eV ascribed to the Co_3_S_4_ (Fig. S10) [44], further confirming the formation of Co_3_S_4_. The C 1*s* spectra shows peaks at ~ 284.5 (C–C), 286.1 (C-N/C-O), and 288.4 (C=O) eV (Fig. 2f), which validates the presence of N,O codoped carbon [9]. The N 1s spectra exhibits three peaks at ~ 400.8, 398.3, and 395.0 eV (Fig. 2g), in which the front two peaks can be assigned to the pyridinic nitrogen, quaternary nitrogen, and pyrrolic nitrogen (Fig. S11), suggesting the existence of N doping carbon [9, 40]. The other peak is ascribed to the Mo 3*p*. The peaks of C–O–Mo at ~ 533.0 eV [9], and C=O at ~ 531.6 eV appear in O 1*s* spectra (Fig. 2h), affirming the existence of O-doped carbon [45]. The presence of C–O–Mo bonds indicates that the monolayer MoS_2_ and carbon matrix are combined by chemical bonding of C–O–Mo [9].

The contents of each element in samples are determined by EA (Table S4). Clearly, carbon mass fraction values are 18.1, 19.2, 20.4, and 21.7 wt%, corresponding to MoS_2_/C, CoMoS_2_/C-I, CoMoS_2_/C-II, and CoMoS_2_/C-III, respectively. After heating theses samples in air atmosphere, Mo, Co, C, and N can be oxidated into MoO_3_, Co_2_O_3_, CO_2_, and NO_2_, respectively, in which the front two result in the increase in the mass; the latter two, together with the loss of O, lead to the decrease in mass. Therefore, the final residues after heating these samples are MoO_3_ and/or Co_2_O_3_. According to the EA results (Table S4), final residues mass for MoS_2_, MoS_2_/C, CoMoS_2_/C-I, CoMoS_2_/C-II, and CoMoS_2_/C-III are 88.9, 73.4, 69.6, 66.2, and 60.1 wt%, respectively, which shows a similar trend with the results in TGA curves (Fig. 2i).

### Lithium-ion Storage Performance Testing in Half Cells

**Fig. 3:**
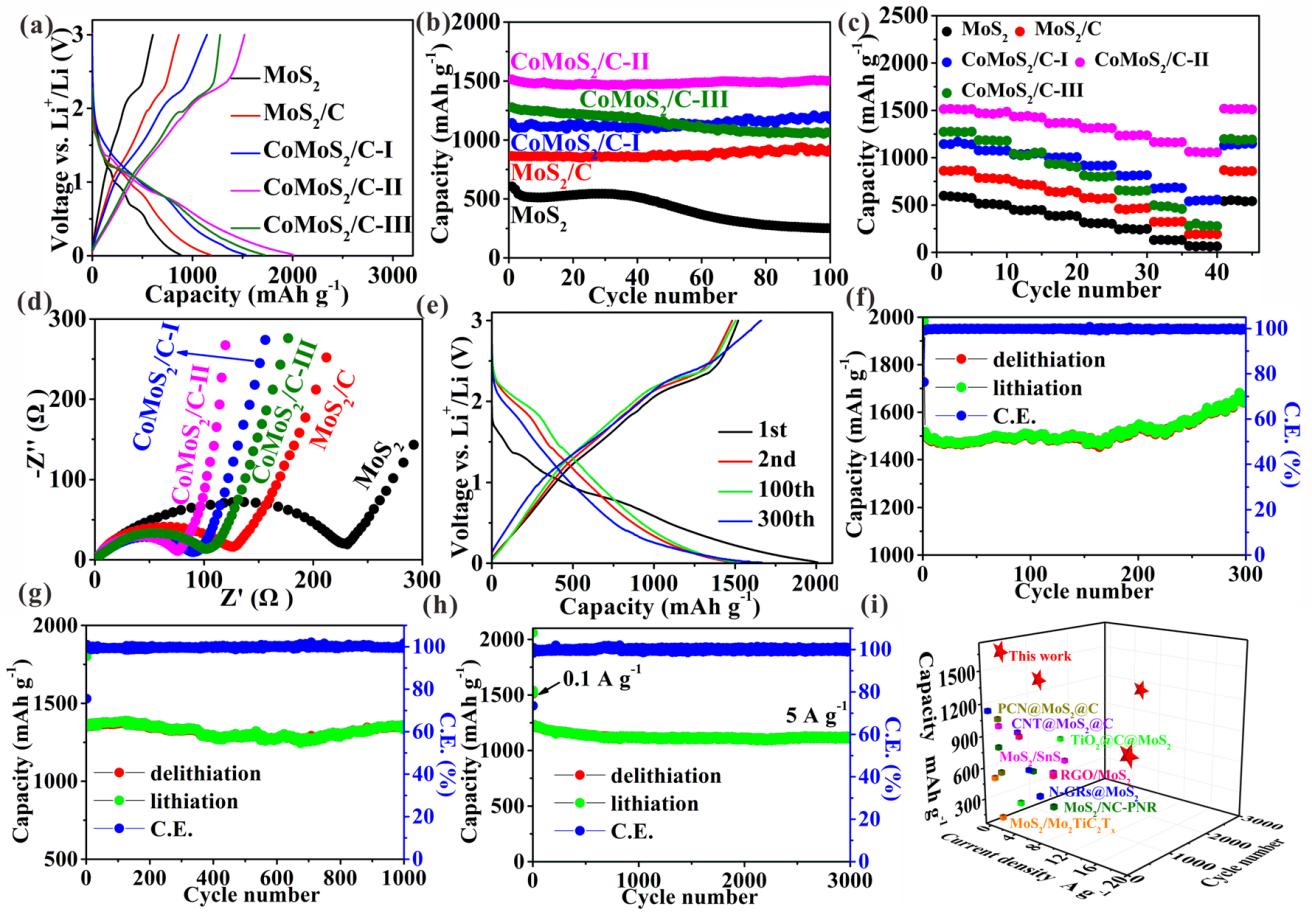
Lithium-ion storage performance testing and comparison. **a** First lithiation/delithiation profiles at 0.1 A g^−1^. **b** Cycling profiles at 0.1 A g^−1^. **c** Rate profiles, and **d** Nyquist plots of all the prepared samples. **e** Lithiation/delithiation profiles at 0.1 A g^−1^. Cycling profiles at **f** 0.1 A g^−1^, **g** 1 A g^−1^, and **h** 5 A g^−1^ of CoMoS_2_/C-II. **i** Performance comparison curve

Figure 3a shows the first charge/discharge curves, in which the CoMoS_2_/C-II shows a higher first capacity (1512.9 mAh g^−1^) than the others (i.e., 605.1 mAh g^−1^ of MoS_2_, 860.3 mAh g^−1^ of MoS_2_/C, 1144.3 mAh g^−1^ of CoMoS_2_/C-I, and 1272.8 mAh g^−1^ of CoMoS_2_/C-III) due to its successful construction of Co-doped monolayer MoS_2_ and the absence of large-size Co_3_S_4_. Figure 3b shows the cycling curves, in which the CoMoS_2_/C-II shows the highest capacity (1504.3 mAh g^−1^) after 100 cycles and delivers almost 100% capacity retention similar to MoS_2_/C and CoMoS_2_/C-I. This is due to the integrity of electrode structure after cycling (Fig. S12) and the low volume expansion in the electrode thickness (11.8%, Fig. S13) after 100 cycles. Obviously, the pure MoS_2_ shows the worst cyclability with a retention of 41.5% after 100 cycles due to its severe structural deterioration (Fig. S12a, b) and large volume expansion in the electrode thickness (116.5%, Fig. S13a, b) after 100 cycles, which is attributed to the absence of few-layered or single-layered MoS_2_ and N,O codoped carbon matrix. Besides, the CoMoS_2_/C-III also shows poor cyclability with a retention of 83.6% after 100 cycles due to electrode cracking (Fig. S12i, j) caused by the presence of large-sized Co_3_S_4_. Figure 3c shows the rate performances of these samples, in which the CoMoS_2_/C-II obviously exhibits superior Li^+^ transport rate (1063.6 mAh g^−1^ at 20 A g^−1^) compared to other samples, which is ascribed to its lowest charge transfer resistance (*R*_ct_, the diameter of semicircle in the high frequency 75.6 Ω**,** data from fitted circuit shown in Fig. S14 and Table S5) [3], the lowest ion diffusion impedance (the slope of inclined line in the low frequency, Fig. 3d and Table S5) [3], the highest specific surface area (Fig. S15 and Table S6), and the highest EC (Table S7). Lower R_ct_ and ion diffusion impedance can accelerate charge transfer, thus strengthening rate capability. Higher specific surface area can ensure more complete contact of active materials and electrolyte to boost ion transport and thus improve the rate performance. Besides, a higher EC can cause electrons to transfer faster, thus enhancing the rate capability.

Due to its best lithium-ion storage performance, the electrochemical behaviors of CoMoS_2_/C-II are further tested. A first charge capacity of 1512.9 mAh g^−1^ with a CE of 75.3% is achieved (Fig. 3e), in which the 24.7% is owing to irreversible solid electrolyte interphase (SEI) film [9]. Besides the first charge/discharge curves, the other curves show a similar shape (Fig. 3e), which indicates high electrode stability upon cycling. From Nyquist plots (Fig. S16), after the first cycle, the R_ct_ increases mostly due to the formation of the SEI film, which decreases with the cycle mainly attributed to the electrochemical activation [10]. After 300 cycles, a high capacity of 1661.6 mAh g^−1^ with a retention of 109.8% is achieved at 0.1 A g^−1^. The capacity increases with increasing number of cycles may be attributed to extensive interfacial storage of Li^+^, amorphization of active materials, and change of Li^+^ storage reaction [46]. Besides, high reversible capacities of 1353.1, 1261.2, and 1115.2 mAh g^−1^ with retentions of 99.5%, 95.9%, and 92.0% are achieved at 1, 2, and 5 A g^−1^ after 1000, 2000, and 3000 cycles (Figs. 3g, S17 and 3 h, respectively). So high capacity retentions demonstrate the superior stability of CoMoS_2_/C-II electrodes under high current densities. The lithium-ion storage performances of CoMoS_2_/C-II are better than that of the MoS_2_-based anode (Fig. 3i and Table S8). Note that the cycling capacity at the low current density of 0.1 A g^−1^ obviously increases with increasing cycling number (Fig. 3f), which is different from other current densities (see Figs. 3g, S17 and 3 h). The phenomena often appear in the transition metal sulfides and oxides [19, 47, 48], which may be attributed to two points: 1) the lithium-ion storage sites of the active materials can be continuously activated during cycling due to the long reaction time at low current density, thus providing a significant increase in capacity upon cycling [48]; 2) pseudocapacitance has a dominant contribution to the total capacity at high current density [48].

**Fig. 4:**
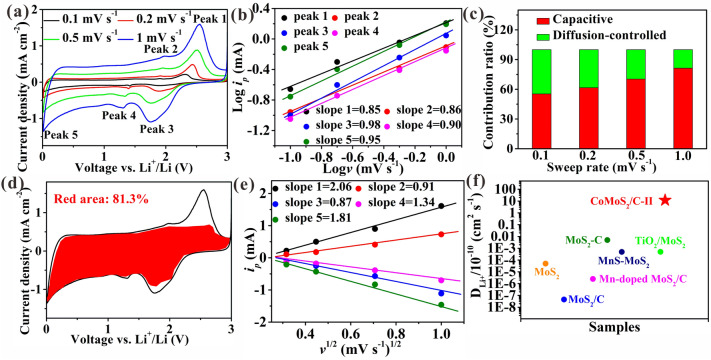
Kinetic analysis of CoMoS_2_/C-II. **a** CV curves. **b** Log*i*_*p*_* vs* Log*v*. **c** Pseudocapacitive contribution percentages. **d** Specific capacitive contribution curve. **e** *i*_*p*_* vs v*^*1/2*^. **f** Comparison of *D*_*Li*+_

**Fig. 5:**
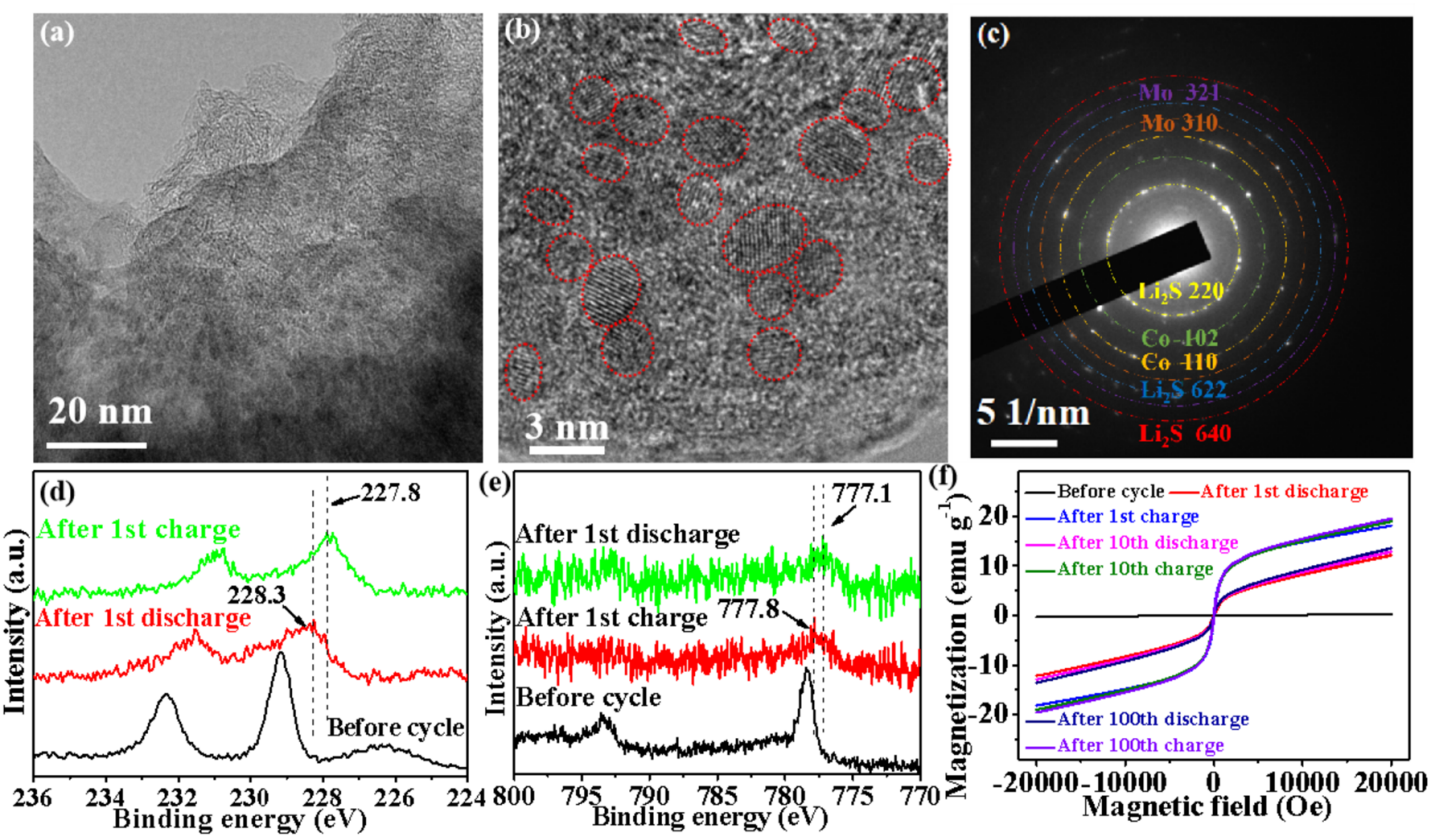
*Ex situ* testing of CoMoS_2_/C-II electrodes. **a**, **b** TEM images after discharging to 0.01 V and **c** corresponding SAED pattern. XPS spectra of **d** Mo 3*d*, **e** Co 2*p*. **f** Magnetic hysteresis loops

The kinetic analysis of the CoMoS_2_/C-II is performed by testing the CV curves at different rates (Fig. 4a). The equation $i = {\text{av}}^{b}$ shows the relationship between current (*i*) and scan rate (*v*) [14]. Generally, the b values of 0.5 and 1.0 indicate the diffusion-controlled and capacitance process, respectively. Clearly, the b values in the redox peaks are about 0.9 (Fig. 4b) to validate the main contribution of pseudocapacity in Li^+^ storage. In addition, the equation $i\left( V \right) = k_{1} v + k_{2} v^{1/2}$ reveals the specific contribution percentages of capacitive and diffusion-controlled behaviors [14], where *i*(*V*), *k*_*1*_*V*, and *k*_*2*_*V*^*1/2*^ represent the total current at a fixed voltage (*V*), pseudocapacitance ratio, and diffusion-controlled ratio, respectively. Obviously, the pseudocapacitance contribution increases from 55.3 to 81.3% (Fig. 4c), corresponding to the scan rate of 0.1 to 1 mV s^−1^, respectively, higher than other samples (i.e., 3.7–14.3% of MoS_2_ (Fig. S18), 28.3–50.3% of MoS_2_/C (Fig. S19), 39.3–65.3% of CoMoS_2_/C-I (Fig. S20), and 43.2–69.0% of CoMoS_2_/C-III (Fig. S21). The higher pseudocapacitance ratio for CoMoS_2_/C-II results in faster ion transport and greater ion storage than other samples. The detailed pseudocapacitance contribution (81.3%) at 1 mV s^−1^ is depicted in red region (Fig. 4d). Note that the peak 5 at 0.01 V does not belong to the reduction peak, which is ascribed to pseudocapacity lithium-ion storage on the surface of formed superparamagnetic Mo and Co particles during conversion reaction. As confirmed in Fig. 5 below, the space charge zone is constructed on the surface of formed superparamagnetic Mo and Co particles after the electrodes discharged to 0.01 V. Due to the formation of space charge zone, rectangular CV curves representing capacitive or pseudocapacitive behavior appear in voltage range of 0.01–1.0 V. The *b* value for peak 5 at 0.01 V is calculated to be approximately equal to 1, which signals that a strong capacitive response occurs in the CoMoS_2_/C-II electrode. The equation $i_{p} = 2.69 \times 10^{5} \,n^{3/2} \,{\text{AD}}_{{{\text{Li}} + }}^{1/2} \,C_{{{\text{Li}} + }} \,v^{1/2}$ can be used to calculate the lithium-ion diffusion coefficient (*D*_Li_^+^) [7] to further reveal Li^+^ diffusion kinetics, in which *i*_*p*_, *v*, *A*, *n*, and *C*_Li+_ represent peak current, scan rate, contact area of materials/electrolyte, electrons number involved in reaction, and Li^+^ bulk concentration, respectively. According to linear relationship of $i_{p}$ and $v^{1/2}$ (Figs. 4e and S18–S21), the calculated *D*_Li_^+^ of CoMoS_2_/C-II is in the range of 7.05 × 10^−10^–1.67 × 10^−9^ cm^2^ s^−1^ with an average value of 1.19 × 10^−9^ cm^2^ s^−1^. This value is higher than 2.21 × 10^−12^, 1.58 × 10^−11^, 2.43 × 10^−10^, and 1.72 × 10^−10^ cm^2^ s^−1^, corresponding to MoS_2_, MoS_2_/C, CoMoS_2_/C-I, and CoMoS_2_/C-III, respectively. The results fully demonstrate that monolayer MoS_2_ has faster lithium-ion diffusion capability than multilayered or few-layered MoS_2_. The *D*_Li+_ of CoMoS_2_/C-II is higher than the value reported for MoS_2_-based anodes (Fig. 4f and Table S9), which is advantageous to fast Li^+^ transport. The high pseudocapacitive ratio is owing to extra lithium-ion storage sites from interfaces, defects, and space charge region, which are confirmed below.

### Characterizations of Electrode before and after Cycling

Space charge zone is confirmed in detail. TEM images (Fig. 5a, b), SAED pattern (Fig. 5c), and EDS mapping images (Fig. S22) validate homogeneous distribution and appearance of ultrasmall Mo and Co particles (~ 2.0 nm, marked by red circles) after the electrode discharged down to 0.01 V. Vast spin-polarized electrons can enter the *d* orbits of Mo and Co under an electric field to trigger spin-polarized surface capacitance, thus creating a space charge region (Fig. S23) [49]. Compared to original Mo 3*d* (229.3 eV) and Co 2*p* (778.5 eV), Mo 3*d* and Co 2*p* (Fig. 5d, e, respectively) show an apparent shift to 228.3 and 777.8 eV belonging to Mo^0^ and Co^0^ after the electrode discharged to 0.01 V. This validates the formed metal Mo and Co [49, 50], which further shifts to 227.8 and 777.1 eV when the electrode is charged to 3 V owing to delithiation of Mo and Co surface, respectively [49]. Additionally, compared to original electrode magnetization of ~ 0 emu g^−1^ (Fig. 5f), a magnetization of 12.3 emu g^−1^ is obtained after the electrode discharged to 0.01 V owing to the formed superparamagnetic Mo and Co particles. This continues to increase to 18.4 emu g^−1^ after the electrode charged to 3 V due to surface delithiation of Mo and Co [49]. Detailly, vast extra electrons storing into Mo and Co partly offset spin majority bands of 4*d* (Mo) and 3*d* (Co) energy levels, thereby causing the magnetization reduction. When Li ions are removed from the surface of Mo and Co, the electrons also are transferred out from the interior of Mo and Co, thus bringing about the increase in magnetization. After charging up to 3 V, the lower binding energy of Mo^0^ and Co^0^ and the higher electrode magnetization are attributed to the irreversibility of Co and Mo. Generally, the first discharging process involves phase transformation reactions of ${\text{MoS}}_{{2}}  ({\text{MoS}}_{{2}} + {\text{xLi}}^{ + } + {\text{xe}}^{ - } \to {\text{Li}}_{{\text{x}}} {\text{MoS}}_{{2}} )$, and the subsequent conversion of Li_x_MoS_2_ to Li_2_S and ${\text{Mo }}\left( {{\text{Li}}_{{\text{x}}} {\text{MoS}}_{{2}} + \left( {{4} - {\text{x}}} \right){\text{Li}}^{ + } + \left( {{4} - {\text{x}}} \right){\text{e}}^{ - } \to {\text{Mo}} + {\text{Li}}_{{2}} {\text{S}}} \right)$ [8, 9, 19, 21, 32]. During the first charging process, Li^+^ is removed from Li_2_S to produce ${\text{S}}\left( {{\text{Li}}_{{2}} {\text{S}} \to {\text{S}} + {\text{2Li}}^{ + } + {\text{2e}}^{ - } } \right)$ [8, 9, 19, 21, 32]. In subsequent cycles, the charging process follows the reaction ${\text{Li}}_{{2}} {\text{S}} \to {\text{S}} + {\text{2Li}}^{ + } + {\text{2e}}^{ - }$, and the discharging process follows the reaction ${\text{S}} + {\text{2Li}}^{ + } + {\text{2e}}^{ - } \to {\text{Li}}_{{2}} {\text{S}}$ [19, 21, 32]. Obviously, the formed Mo in the first charging process is irreversible. Similarly, Co is also irreversible. Clearly, in the following cycles, the electrode magnetization is basically unchanged (Fig. 5f) compared with the first charging/discharging, which confirms that the space charge region has a persistent effect. These results confirm the successful creation of space charge region when Mo and Co nanoparticles are formed.

To further demonstrate the advantage of monolayer MoS_2_ on lithium-ion storage, *ex situ* TEM observations are performed after discharging the MoS_2_, MoS_2_/C, CoMoS_2_/C-I, and CoMoS_2_/C-III electrodes to 0.01 V, where nanoparticles with sizes of ~ 5.4, 4.0, 3.0, and 3.5 nm are formed for pure MoS_2_ (Fig. S24), MoS_2_/C (Fig. S25), CoMoS_2_/C-I (Fig. S26), and CoMoS_2_/C-III (Fig. S27) electrodes, respectively. Obviously, the size (~ 2 nm) of nanoparticles obtained for CoMoS_2_/C-II electrode after discharging to 0.01 V (Fig. 5b) is the smallest among these samples due to the limited two-dimensional transport of Mo and Co atoms in the monolayer [21] and the absence of large-sized Co_3_S_4_. The smaller nanoparticles have a larger specific surface area to produce more interfaces for storing Li^+^ [46, 51] and create a stronger space charge region [49, 52], thus heightening the capacity and Li^+^ transport rate.

Considering the above results, the capacity of CoMoS_2_/C-II is about 2.5 times theoretical capacity of MoS_2_ for the following reasons: i) enormous Co doping sites to enhance surface energy of MoS_2_ to store extra Li^+^ [7, 19]; ii) extremely high capacity of monolayer MoS_2_ [30, 32]; iii) vast contact interfaces of monolayer MoS_2_ and C to increase Li^+^ active sites [7, 9, 46, 51]; iv) formed ultrasmall Co particles to create strong space charge region as excess Li^+^ active sites [49, 52]. Among them, creating space charge regions during conversion reaction due to the formation of ultrasmall Co has changed Li^+^ storage mechanism of traditional MoS_2_ materials.

### Lithium-Ion storage Performances in Full Cells

**Fig. 6:**
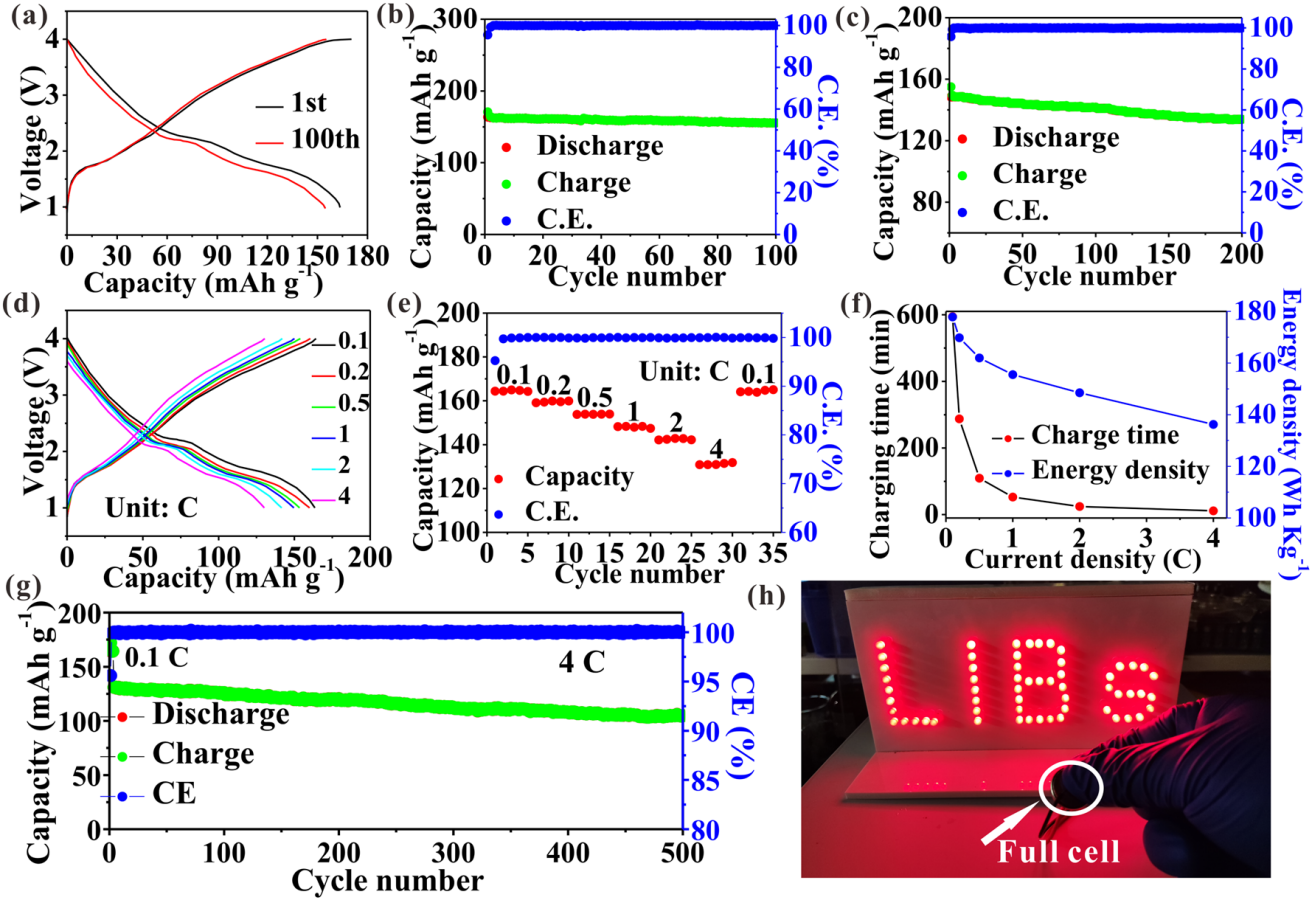
Full cells testing. **a** Voltage profiles at 0.1 C. **b** Cycling curves at 0.1 C. **c** Cycling curves at 1 C. **d**, **e** Rate curves. **f** Charging time and corresponding *E*_*G*_. **g** Cycling curves at 4 C. **h** Lighting LED

Full cells assembled with a commercial LiFePO_4_ cathode are tested to evaluate the application potential of CoMoS_2_/C-II anode. The charge/discharge profiles of cycles 1 and 100 (Fig. 6a) show a similar shape, indicating excellent stability of the electrode in cycles, in which a nominal voltage of ~ 2.2 V and a high capacity of 164.4 mAh g^−1^ with 95.1% retention are achieved at 0.1 C after cycling (Fig. 6b). Moreover, a high capacity of 133.9 mAh g^−1^ is obtained after cycling at 1 C with a high retention of 90.2% (Fig. 6c). Figure 6d, e exhibits rate curves where high capacities of 164.3, 159.9, 153.9, 148.4, 142.3, and 131.8 mAh g^−1^ are obtained at 0.1, 0.2, 0.5, 1, 2, and 4 C, respectively. Equation $\left( {E_{G} ,{\text{Wh kg}}^{{ - {1}}} } \right) = \left( {C_{c} \times V} \right)/\left( {m_{{{\text{active}}}} + m_{{{\text{inactive}}}} } \right)$ [10] can be used to calculate the gravimetric energy density (*E*_*G*_), where *C*_*c*_, *V, m*_active_, and *m*_inactive_ denote the cell capacity (2.7 mAh), nominal voltage, active mass of cathode and anode (17.8 mg), and inactive mass (15.6 mg), respectively. The *E*_*G*_ at 0.1 C can be calculated as 177.8 Wh kg^−1^ and remains 136.2 Wh kg^−1^ (76.6% retention relative to 177.8 Wh kg^−1^) at 4 C within a charging time of 11.5 min (Fig. 6f). After activation at 0.1 C, the full cell retains a high retention of 80.2% after 500 cycles at 4 C (Fig. 6g). The above results fully imply excellent rate performances and cycling stability of the cell, which are superior to previously reported cells of MoS_2_-based anode materials (Table S10). Clearly, a light-emitting-diode array with 59 unit elements is cushily lighted by a single full cell (Fig. 6h) and the lighting time is about 2 h. The above results fully imply that the CoMoS_2_/C-II materials have potential practicality for fast-charging LIBs with high energy density.

### Effect of Co on Monolayer MoS_2_ Revealed by DFT

**Fig. 7:**
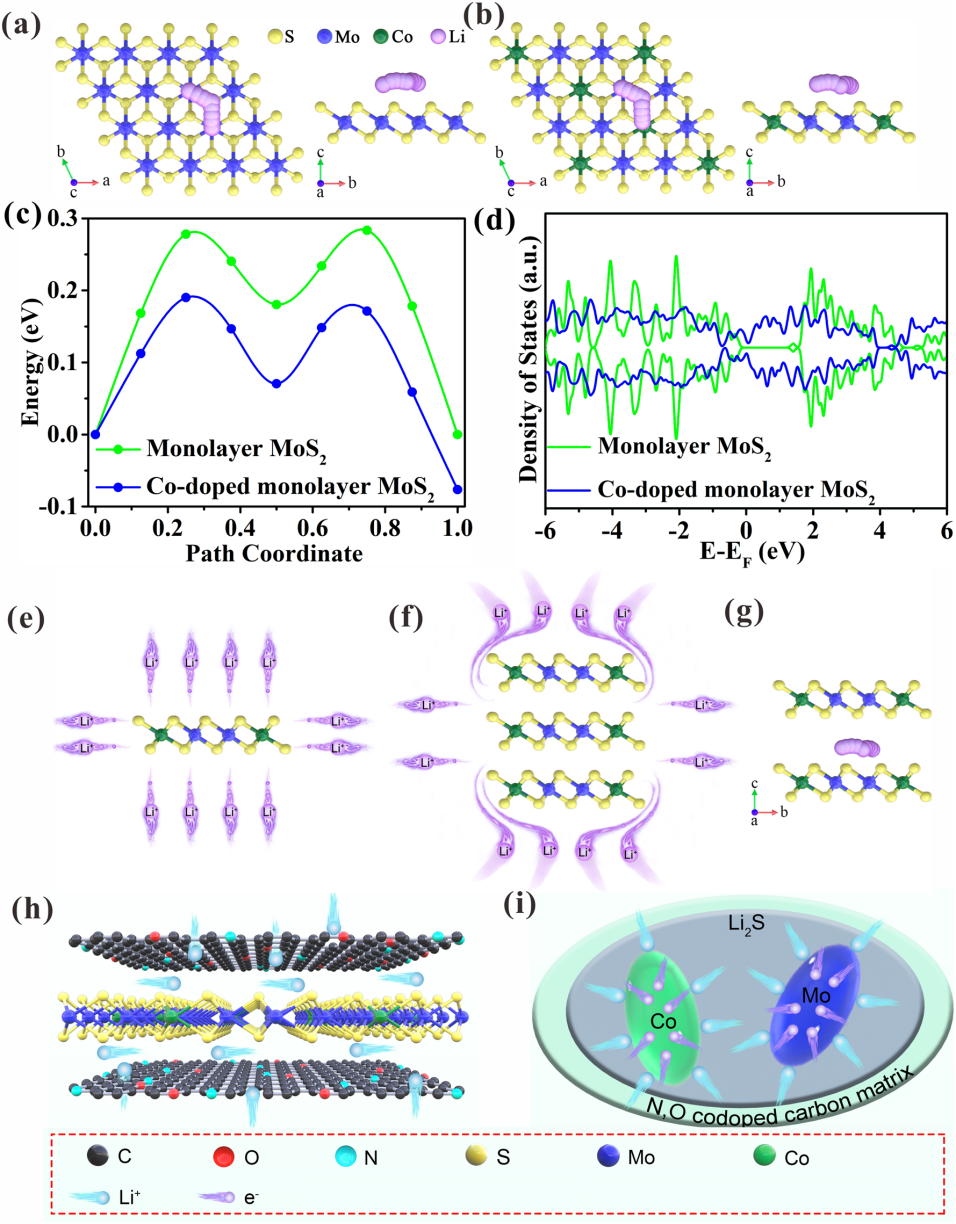
Configurations of Li^+^ migration paths in **a** monolayer MoS_2_, **b** Co-doped monolayer MoS_2_, **c** Li^+^ diffusion energy barrier. **d** Calculated DOS. Schematic diagram of lithium ion **e** isotropic transport in monolayer MoS_2_, and **f** anisotropic transport in multilayered MoS_2_. **g** Configurations of Li^+^ migration paths in multilayered MoS_2_. **h**, **i** Diagrammatic sketch of Li^+^ storage mechanism in CoMoS_2_/C-II

To investigate the role of Co doping on energy barriers of lithium-ion diffusion in the monolayer MoS_2_ plane of CoMoS_2_/C-II, the DFT calculations are performed, where the amount of Co doping follows the XPS results (Table S2). Figure 7a, b is configurations of Li^+^ migration paths in monolayer MoS_2_ and Co-doped monolayer MoS_2_, respectively. It can be seen that the Li-ion diffusion energy barrier (0.19 eV) of Co-doped monolayer MoS_2_ is lower than 0.28 eV of monolayer MoS_2_. This implies that Co doping can greatly improve the Li-ion diffusion kinetics, thus causing fast Li^+^ transport. Besides, the doped Co atoms can significantly reduce the bandgap of monolayer MoS_2_ to enable high carrier transport, as validated by calculated density of states (DOS) (Fig. 7d). Monolayer MoS_2_ shows a semiconductor attribute with a bandgap of 1.30 eV, while the Co-doped monolayer MoS_2_ exhibits a metallic characteristic with a bandgap of 0 eV to improve carrier transport due to the lattice distortion of MoS_2_ triggered by Co doping, thereby adjusting the electronic structure of MoS_2_ [53, 54].

Besides, the fully exposed basal plane for Co-doped monolayer MoS_2_ can make lithium ions have isotropic transport behavior (Fig. 7e). Evidently, the multilayered MoS_2_ has anisotropic transport behavior (Fig. 7f). To support this view, the corresponding DFT calculations are executed. Lithium ions transport to react with multilayered structure including A, B, and C layers shown in Fig. S28. Li^+^ diffuses to react with B and C layers, which involves two diffusion paths: path I is completely through layer A (Fig. S28a); path II is to bypass layer A and diffuse to the interlayer to react with layers B and C (Fig. S28b). Through DFT calculation, it is found that path I is impossible because forcing lithium-ion diffusion through layer A makes the model collapse. In other words, the diffusion energy barrier that lithium ion in path I needs to overcome is extremely high. Path II is feasible, and DFT calculation results show that the diffusion energy barrier of lithium ion between layers is only 0.23 eV (Figs. 7g and S29). This indicates that lithium ions preferentially choose interlayer transport to confirm anisotropic Li^+^ transport characteristics of multilayered structure. Once multiple layers are reduced to single layer, which can cause the disappearance of interlayer structure, thereby transforming anisotropic Li-ion storage into isotropic Li-ion storage.

In view of the above results, the CoMoS_2_/C-II shows outstanding electrochemical performances, especially fast-charging performances, which are mainly attributed to its unique nanostructure. First, the Co-doped monolayer MoS_2_ with fully exposed basal planes can maximize the contact area with the amorphous carbon matrix, which not only transforms lithium-ion transport path from anisotropy to isotropy, but also substantially improves the EC of monolayer MoS_2_, thus enhancing lithium-ion transport (Fig. 7e). Second, monolayer MoS_2_, together with the carbon matrix, can fully limit the growth of Co and Mo to obtain very small nanoparticles (~ 2 nm) during conversion reaction, thus creating strong surface-capacitance effects to accelerate lithium-ion transport (Fig. 7f).

## Conclusions

An original nanocomposite consisting of Co-doped monolayer MoS_2_ and N,O codoped carbon substrate is successfully fabricated via in situ construction of electrostatic repulsion in the MoS_2_ interlayer under high-pressure vapor phase. DFT calculation results demonstrate the formation mechanism of monolayer MoS_2_ and confirm that the bandgap and lithium-ion diffusion energy barrier of monolayer MoS_2_ can be reduced by Co doping, thus boosting the charge transfer. Besides, monolayer MoS_2_ possesses an open lithium-ion transport path and a maximum contact area with the carbon matrix to achieve high D_Li+_. In addition, the doped Co atoms can create a space charge region during the conversion reaction, thus accelerating the ion transport rate. In view of these advantages, the nanocomposite exhibits an ultrahigh capacity of 1661.6 mAh g^−1^ and an ultrafast ion transport capability of 1063.3 mAh g^−1^ at 20 A g^−1^. Besides, a high energy density of 136.2 Wh kg^−1^ is obtained at 4 C in a charging time of 11.5 min in full cells, indicating that the fabricated nanocomposite is a promising fast-charging anode in LIBs.

### Supplementary Information

Below is the link to the electronic supplementary material.

Supplementary file1 (PDF 3002 KB)
