# Supplementary material for: Monolayer MoS2 Fabricated by In Situ Construction of Interlayer Electrostatic Repulsion Enables Ultrafast Ion Transport in Lithium-Ion Batteries
Source: Nanomicro Lett. 2023 Mar 31;15:80. doi: 10.1007/s40820-023-01042-4 (PMC10066056; doi:10.1007/s40820-023-01042-4)
Supplement: Supplementary file 1 — Supplementary file1 (PDF 3002 KB) [file 40820_2023_1042_MOESM1_ESM.pdf]

Supporting Information for

Monolayer MoS_2 Fabricated by In-Situ Construction of Interlayer Electrostatic Repulsion Enables Ultrafast Ion Transport in Lithium-Ion Batteries

Meisheng Han[1], Yongbiao Mu[1], Jincong Guo[1], Lei Wei[1], Lin Zeng[1], and Tianshou Zhao[1, *]

[1]Shenzhen Key Laboratory of Advanced Energy Storage, SUSTech Energy Institute for Carbon Neutrality, Department of Mechanical and Energy Engineering, Southern University of Science and Technology, Shenzhen 518055, P. R. China

*Corresponding author. E-mail: zhaots@sustech.edu.cn (Tianshou Zhao)

ORCID: 0000-0003-4825-2381 (Tianshou Zhao)

Supplementary Figures and Tables

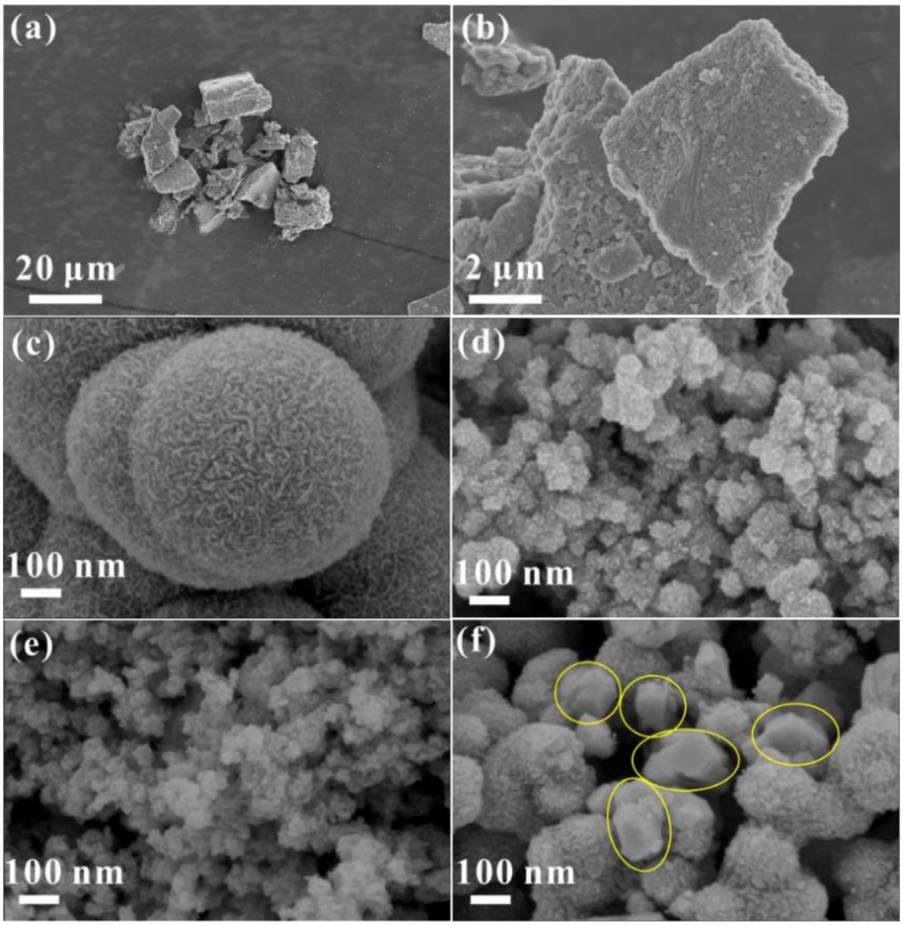

Fig. S1 SEM images of MoS_2 (**a**, **b**), MoS_2/C (**c**), $CoMoS_2$/C-I (**d**), $CoMoS_2$/C-II (**e**), and $CoMoS_2$/C-III (**f**)

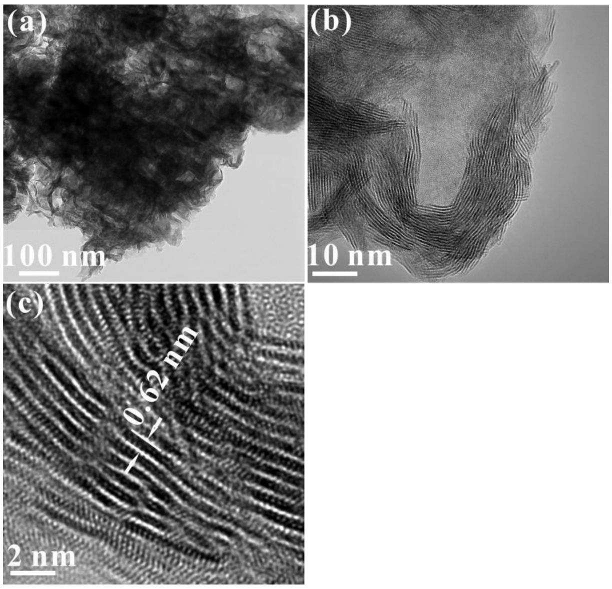

Fig. S2 (**a-c**) TEM images of MoS_2

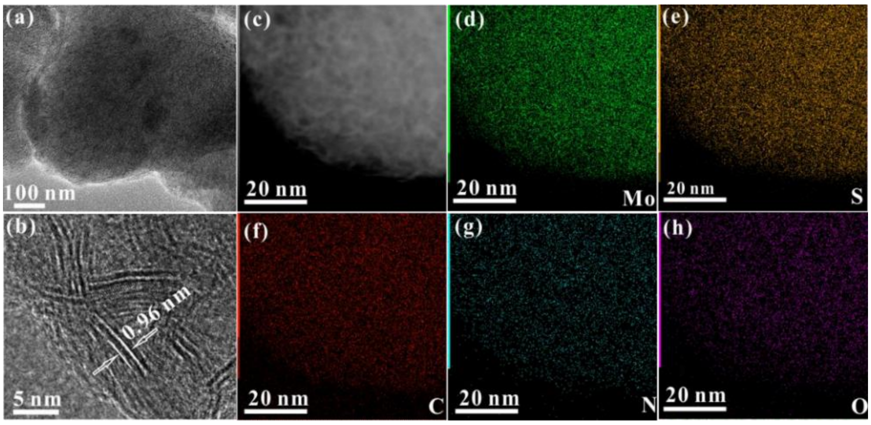

Fig. S3 (**a**, **b**) TEM images, (**c**) HAADF image and corresponding EDS mapping images of Mo (**d**), S (**e**), C (**f**), N (**g**), and O (**h**) of MoS_2/C

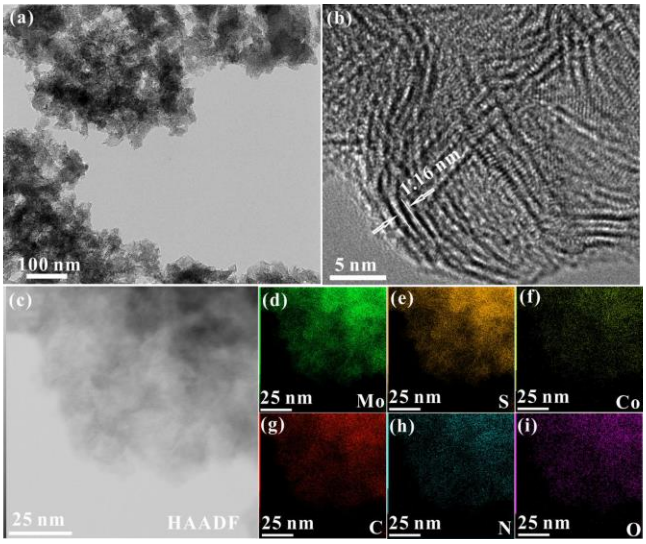

Fig. S4 (**a**, **b**) TEM images, (**d**) HAADF image and corresponding EDS mapping images of Mo (**e**), S (**f**), Co (**g**), C (**h**), N (**i**), and O (**j**) of $CoMoS_2$/C-I

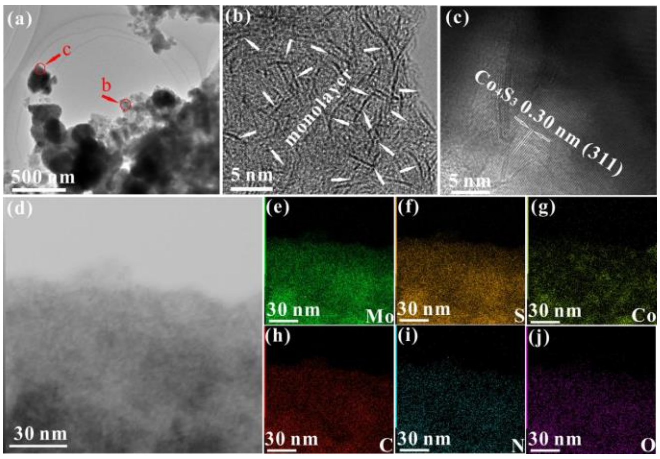

Fig. S5 (**a-c**) TEM images, (**d**) HAADF image taken from red circle pointed by a red arrow b and corresponding EDS mapping images of Mo (**e**), S (**f**), Co (**g**), C (**h**), N (**i**), and O (**j**) of $CoMoS_2$/C-III. Image (**b**) is taken from red circle pointed by a red arrow b in image (**a**). Image (**c**) is taken from red circle pointed by red arrow c in image (**a**)

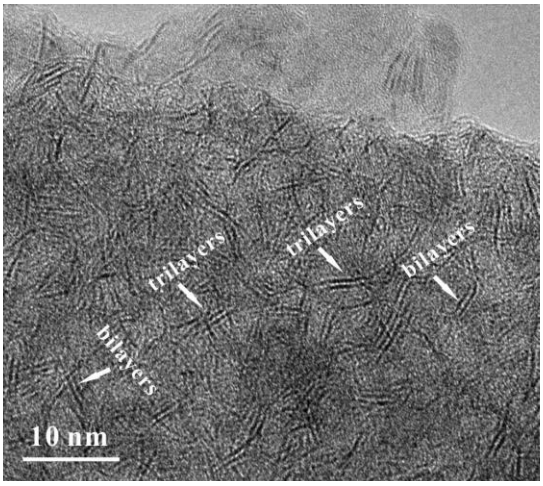

Fig. S6 TEM image of sample obtained in 1.2 g total mass of precusor with a mass ratio (5:4:7) of Cobalt bis (2-ethylhexanoate)/$NH_4)_2MoS_4$/DMF

Obviously, the sample obtained in low total mass of precursor shows few-layerd MoS_2 nanostructure.

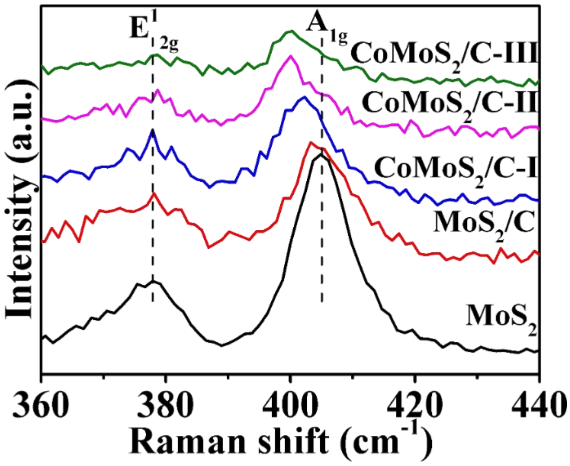

Fig. S7 Raman spectra in the range of 360-440 cm^{-1} of the obtained samples

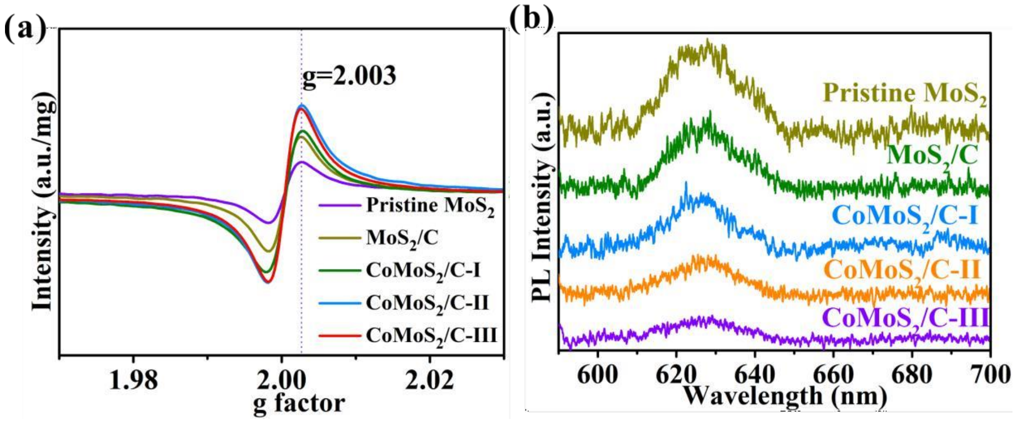

Fig. S8 (**a**) ESR spectra and (**b**) PL spectra of pristine MoS_2, MoS_2/C, and $CoMoS_2$/C samples

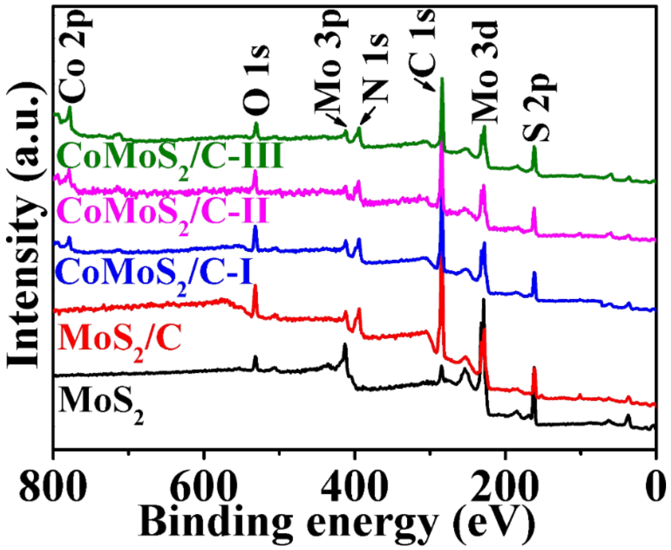

Fig. S9 XPS survey peaks of the obtained samples

Table S1 Fitting results of XPS spectra of all the samples

**Samples**	**Mo (at%)**	**S (at%)**	**Co (at%)**	**C (at%)**	**N (at%)**	**O (at%)**
**MoS2**	31.37	63.05	0.00	4.14	0.00	1.44
**MoS2/C**	5.63	11.50	0.00	70.16	7.95	4.76
**CoMoS2/C-I**	4.49	11.08	0.96	72.40	7.02	4.05
**CoMoS2/C-II**	3.33	10.66	1.82	74.61	5.92	3.66
**CoMoS2/C-III**	1.76	10.27	3.33	76.43	5.09	3.13

The doping amount of N and O elements in the carbon materials should be calculated by N (at%)/(C (at%)+N (at%)+O (at%))*100% and O (at%)/(C (at%)+N (at%)+O (at%))*100%, respectively. The doping amount of Co elements in the MoS_2 materials should be calculated by Co (at%)/(Co (at%)+Mo (at%)+S (at%))*100%, respectively. The specific doping amount is represented in Table S2 and S3.

Table S2 The doping amount of Co elements in the MoS_2 materials

Samples	Co (at%)
**CoMoS2/C-I**	5.80
**CoMoS2/C-II**	11.52
**CoMoS2/C-III**	21.69

Table S3 The doping amount of N and O elements in the carbon materials

Samples	N (at%)	O (at%)
**MoS2/C**	9.59	5.74
**CoMoS2/C-I**	8.41	4.85
**CoMoS2/C-II**	7.03	4.35
**CoMoS2/C-III**	6.01	4.53

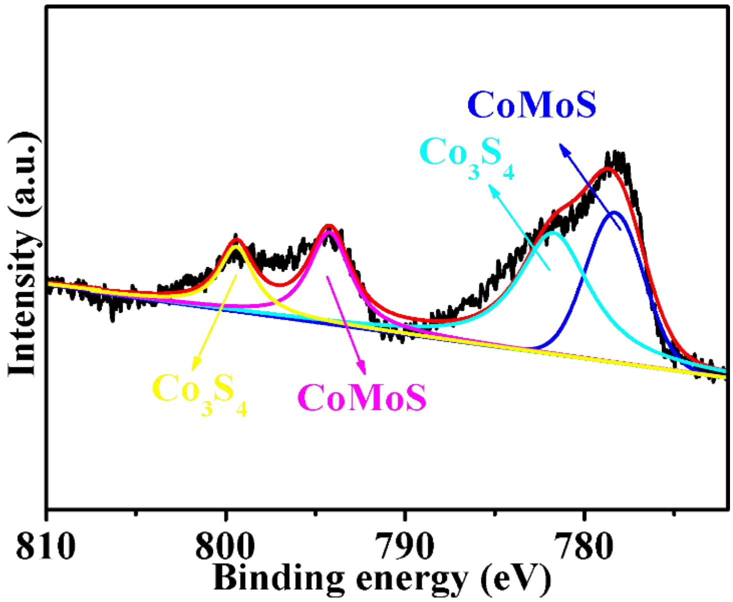

Fig. S10 The fitted spectra of Co 2p of $CoMoS_2$/C-III

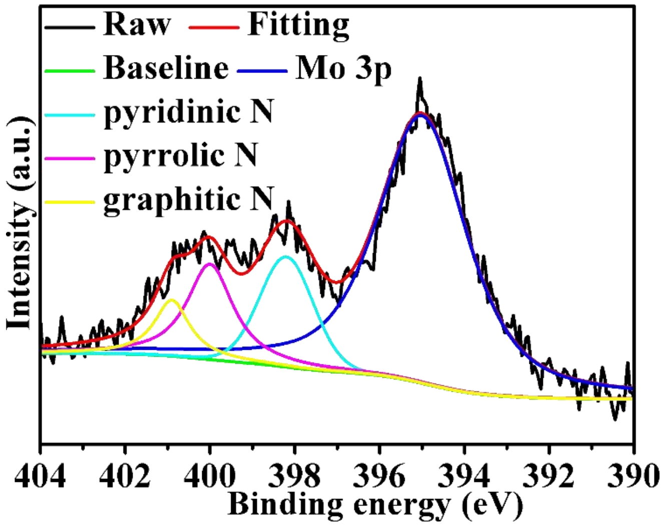

Fig. S11 The fitted spectra of N 1s of $CoMoS_2$/C-II

Table S4 The elemental analysis results of the obtained samples

Samples	Co (wt%)	C (wt%)	N (wt%)	O (wt%)	Mo (wt%)	S (wt%)
**MoS2**	0.0	0.3	0.0	0.2	59.3	40.2
**MoS2/C**	0.0	14.8	1.9	1.4	49.1	32.8
**CoMoS2/C-I**	5.4	16.2	1.8	1.2	41.3	34.1
**CoMoS2/C-II**	12.2	17.7	1.6	1.1	32.6	34.8
**CoMoS2/C-III**	22.1	19.3	1.4	1.0	19.3	36.9

The C, N, O, and S contents in the composites were measured using O/N/H and C/S elemental analyzers. The Co contents in the composites were tested by inductively coupled plasma mass spectrometer. The content of Mo was calculated as a difference to 100 wt%. It can be seen that the mass percentages of N, O codoped carbon matrix are 18.1, 19.2, 20.4, and 21.7wt%, corresponding to MoS_2/C, $CoMoS_2$/C-I, $CoMoS_2$/C-II, and $CoMoS_2$/C-III, respectively.

After heating in air atmosphere, the increase of the mass is ascribed to the oxidation of Mo and Co into MoO_3 and Co_2O_3, while the decrease of the mass is attributed to the oxidation of C, N into CO_2 and NO_2 and the mass loss of O. Consequently, the final residual products are MoO_3 and/or Co_2O_3.

According to the element analysis results (Table S4), the mass of the final residual products for pure MoS_2, MoS_2/C, $CoMoS_2$/C-I, $CoMoS_2$/C-II, and $CoMoS_2$/C-III should be 88.9, 73.4, 69.6, 66.2, and 60.1wt%, respectively.

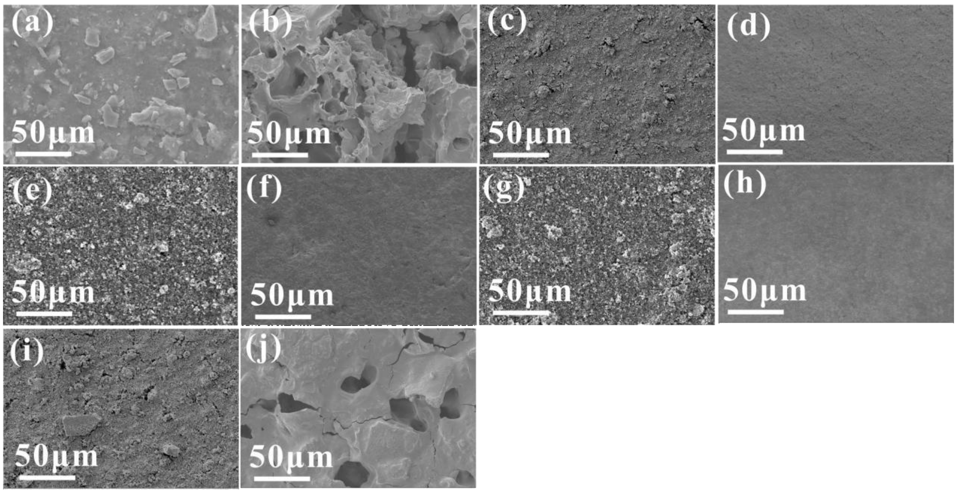

Fig. S12 SEM images of electrode structure before and after cycling. (**a**, **b**) pure MoS_2 before cycling (**a**) and after 100 cycling (**b**); (**c**, **d**) MoS_2/C before cycling (**c**) and after 100 cycling (**d**); (**e**, **f**) $CoMoS_2$/C-I before cycling (**e**) and after 100 cycling (**f**); (**g**, **h**) $CoMoS_2$/C-II before cycling (**g**) and after 100 cycling (**h**); (**i**, **j**) $CoMoS_2$/C-III before cycling (**i**) and after 100 cycling (**j**)

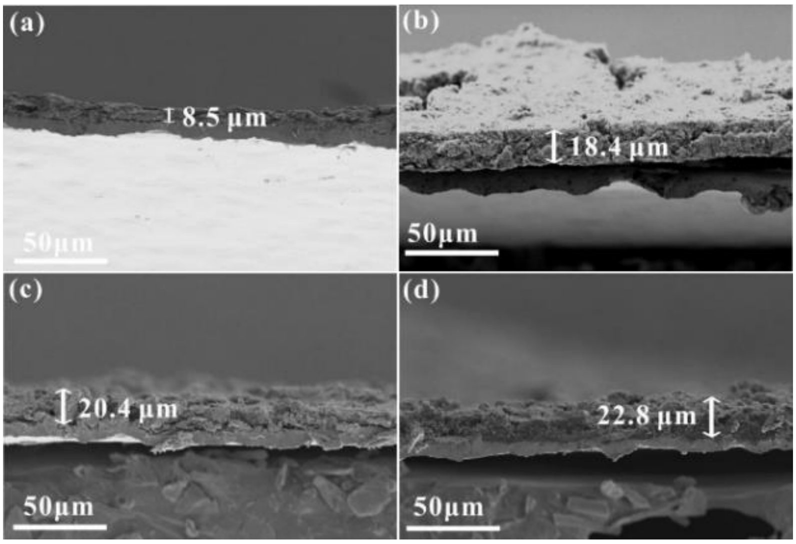

Fig. S13 Cross-sectional SEM images of electrode before and after cycling. (**a**, **b**) pure MoS_2 before cycling (**a**) and after 100 cycling (**b**); (**c**, **d**) $CoMoS_2$/C-II before cycling (**c**) and after 100 cycling (**d**)

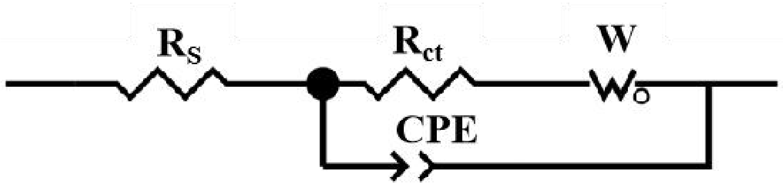

Fig. S14 Fitted circuit of Fig. 3d in the manuscript

Table S5 R_{ct} in the high frequency (data from fitted circuit in Fig. S14) and the slope of inclined line in the low frequency of the obtained samples in the Fig. 3d

Samples	Rct (Ω)	The slope of inclined line in the low frequency
**MoS2**	228.6	2.0
**MoS2/C**	126.7	3.4
**CoMoS2/C-I**	90.2	4.8
**CoMoS2/C-II**	75.6	9.0
**CoMoS2/C-III**	102.8	4.4

The larger slope of inclined line in the low frequency represents lower ion diffusion impedance.

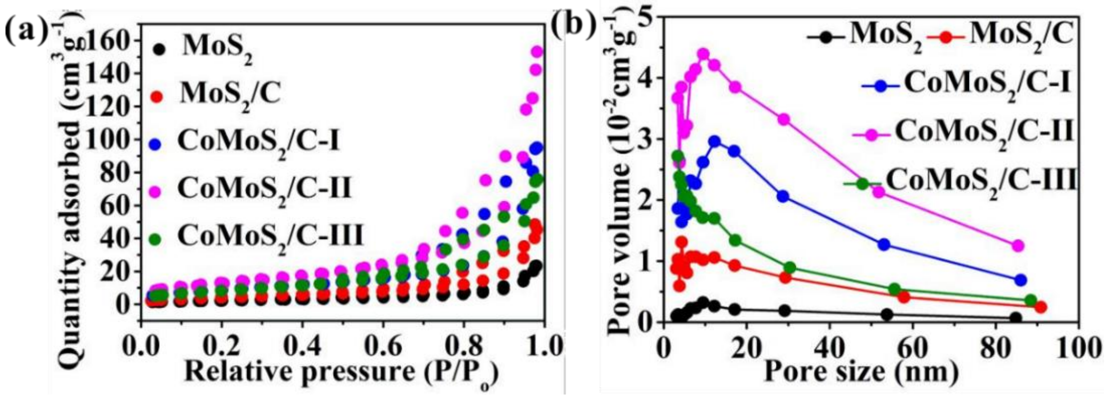

Fig. S15 (**a**) Nitrogen adsorption/desorption isotherms and (**b**) pore size distribution of the obtained samples

Table S6 The specific surface area of the obtained samples

Samples	Specific surface area (m^2 g^{-1})
**MoS2**	6.3
**MoS2/C**	25.7
**CoMoS2/C-I**	63.4
**CoMoS2/C-II**	91.8
**CoMoS2/C-III**	48.6

Higher specific surface area can ensure more fully contact between active materials and electrolyte, thus boosting the lithium-ion transport.

Table S7 The EC of the obtained samples

**Samples**	**Electrical conductivity (S cm^{-1})**
**MoS_2**	1.2×10^{-3}
**MoS_2/C**	4.6
**CoMoS_2/C-I**	23.4
**CoMoS_2/C-II**	56.3
**CoMoS_2/C-III**	18.5

The higher EC is more favorable for charge transport.

EC measurements

First, the obtained powders were added in a hollow cylinder mould with two electrodes on each ends, which were connected with a digital multimeter (Keithley 2001, USA) and followed by compressing powders into slices. During compressing the electrical resistance was observed constantly. When the electrical resistance kept stable the mould was opened, obtaining slices. Subsequently, the EC of slices was measured using a four-probe tester (Probes Tech RTS-8, China).

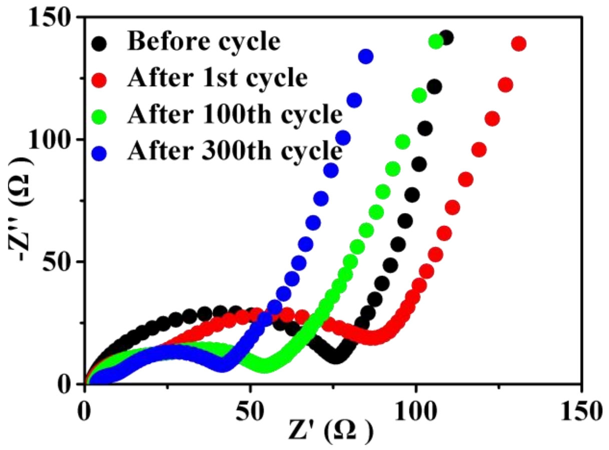

Fig. S16 Nyquist plots of CoMoS_2/C-II at different cycling number

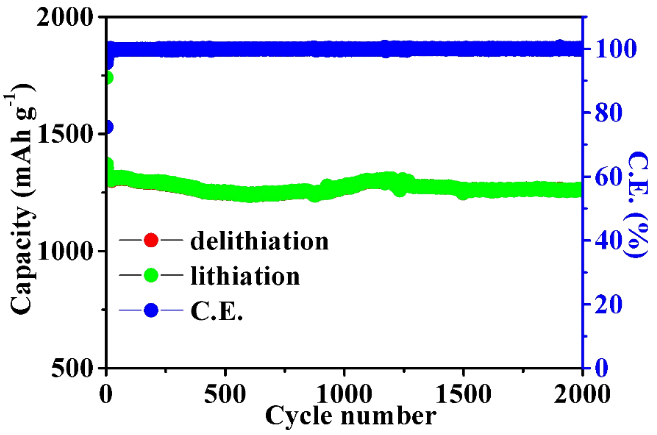

Fig. S17 Cycling performance of CoMoS_2/C-II at 2 A g^{-1}

Table S8 Electrochemical performances of MoS_2-based materials for LIBs anodes in open reports. C_C-final charge capacity (mAh g^{-1}), C_R-capacity retention (%), M_L-mass loading (mg cm^{-2}), J-current density (A g^{-1}), N_C-cycle number, NA-not available

Samples	CC	CR	ML	J	NC	References
**CoMoS2/C-II**	**1661.6**	**109.8**	**1.5**	**0.1**	**300**	**This work**
**CoMoS2/C-II**	**1353.1**	**99.5**	**1.5**	**1**	**1000**	**This work**
**CoMoS2/C-II**	**1261.2**	**95.9**	**1.5**	**2**	**2000**	**This work**
**CoMoS2/C-II**	**1115.2**	**92.0**	**1.5**	**5**	**3000**	**This work**
**CoMoS2/C-II**	**1063.3**	**NA**	**1.5**	**20**	**NA**	**This work**
TiO2@C@MoS2	1150.0	NA	0.5	0.1	NA	Chem. Eng. J. **416**, 129094 (2021)
TiO2@C@MoS2	720	90	2	1	1500	Chem. Eng. J. **416**, 129094 (2021)
TiO2@C@MoS2	379.0	NA	0.5	5	NA	Chem. Eng. J. **416**, 129094 (2021)
N-GRs/MoS2	1151	NA	1	0.1	NA	Chem. Eng. J. **408**, 127269 (2021)
N-GRs/MoS2	547	92.6	1	2	600	Chem. Eng. J. **408**, 127269 (2021)
N-GRs/MoS2	499.3	NA	1	8	NA	Chem. Eng. J. **408**, 127269 (2021)
MoS2/SnS	988	100	NA	0.2	200	Angew. Chem., Int. Ed. **59**, 14621 (2020)
MoS2/SnS	634	53	NA	5	1000	Angew. Chem., Int. Ed. **59**, 14621 (2020)
MoS2/SnS	745	NA	NA	10	NA	Angew. Chem., Int. Ed. **59**, 14621 (2020)
PCN@MoS2@C	1052.5	88%	1.5	0.1	200	Nano Energy **65**, 104061 (2019)
PCN@MoS2@C	609	NA	1.5	2	NA	Nano Energy **65**, 104061 (2019)
MoS2/NC-PNR	800	~96.4	0.7	0.5	150	J. Mater. Chem. A **7**, 7553 (2019)
MoS2/NC-PNR	520	NA	0.7	2	700	J. Mater. Chem. A **7**, 7553 (2019)
MoS2/NC-PNR	443	NA	0.7	10	NA	J. Mater. Chem. A **7**, 7553 (2019)
MoS2/Mo2TiC2Tx	509	91.88	NA	0.1	100	Angew. Chem., Int. Ed. **57**, 1846 (2018)
MoS2/Mo2TiC2Tx	182	NA	NA	2	NA	Angew. Chem., Int. Ed. **57**, 1846 (2018)
CNT@MoS2@C	905	NA	1.00	1	500	Adv. Energy Mater. **8**, 1700174 (2018)
RGO/MoS2	892	93.89	0.41	2	400	Energy Stor. Mater. **10**, 282 (2018)
RGO/MoS2	723	NA	0.41	10	NA	Energy Stor. Mater. **10**, 282 (2018)

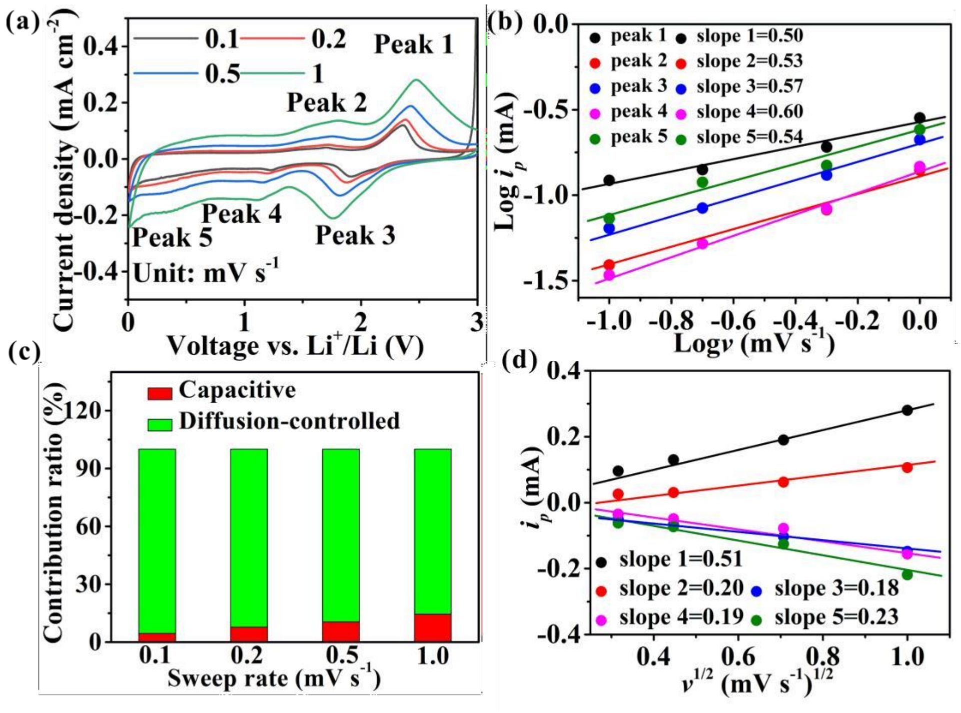

Fig. S18 (**a**) CV curves at different sweep rates, (**b**) Log i_p against Log v at peaks 1-5, (**c**) the percentages of pseudocapacitive contribution at different sweep rates, and (**d**) i_p versus $v^{1/2}$ at peaks 1-5 of MoS_2

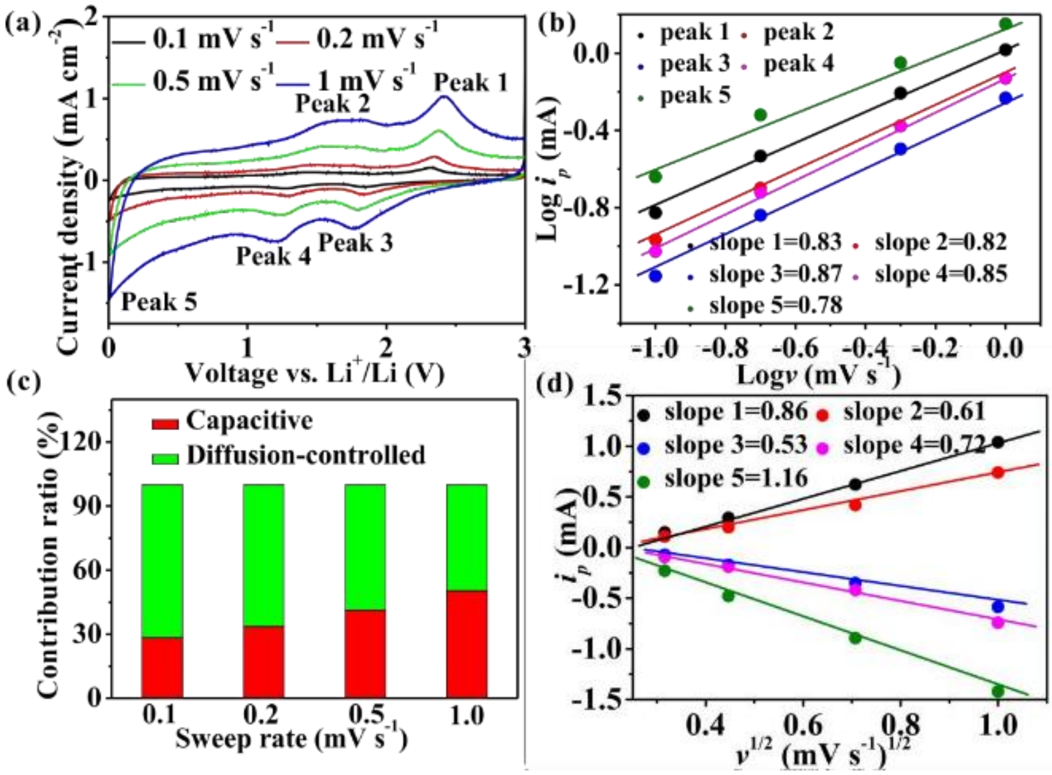

Fig. S19 (**a**) CV curves at different sweep rates, (**b**) Log i_p against Log v at peaks 1-5, (**c**) the percentages of pseudocapacitive contribution at different sweep rates, and (**d**) i_p versus $v^{1/2}$ at peaks 1-5 of MoS_2/C

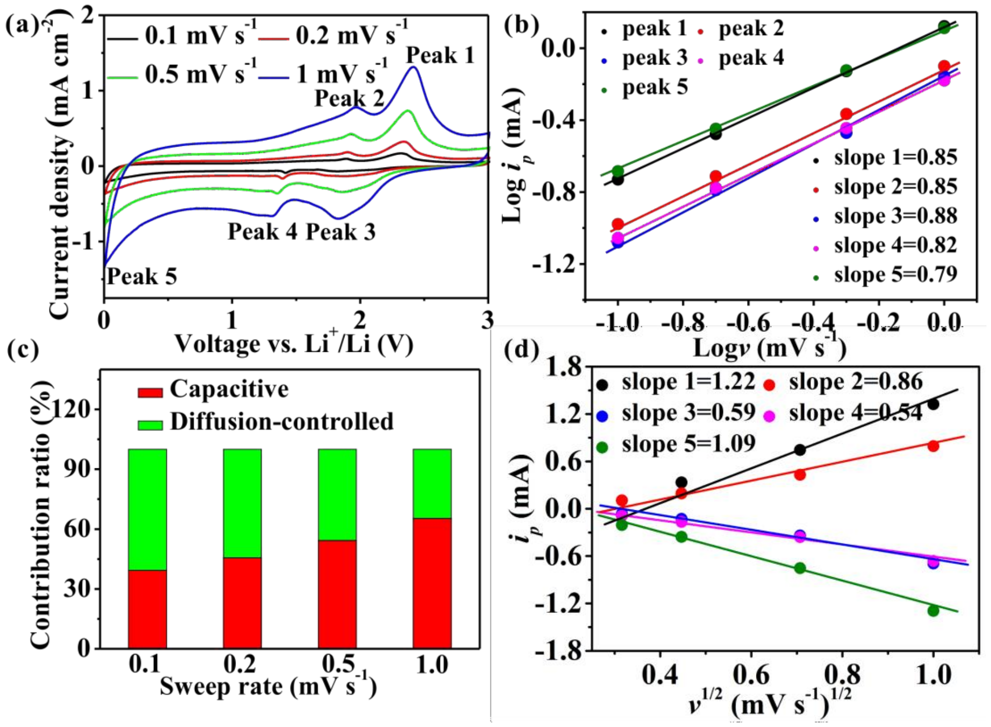

Fig. S20 (**a**) CV curves at different sweep rates, (**b**) Log i_p against Log v at peaks 1-5, (**c**) the percentages of pseudocapacitive contribution at different sweep rates, and (**d**) i_p versus $v^{1/2}$ at peaks 1-5 of $CoMoS_2$/C-I

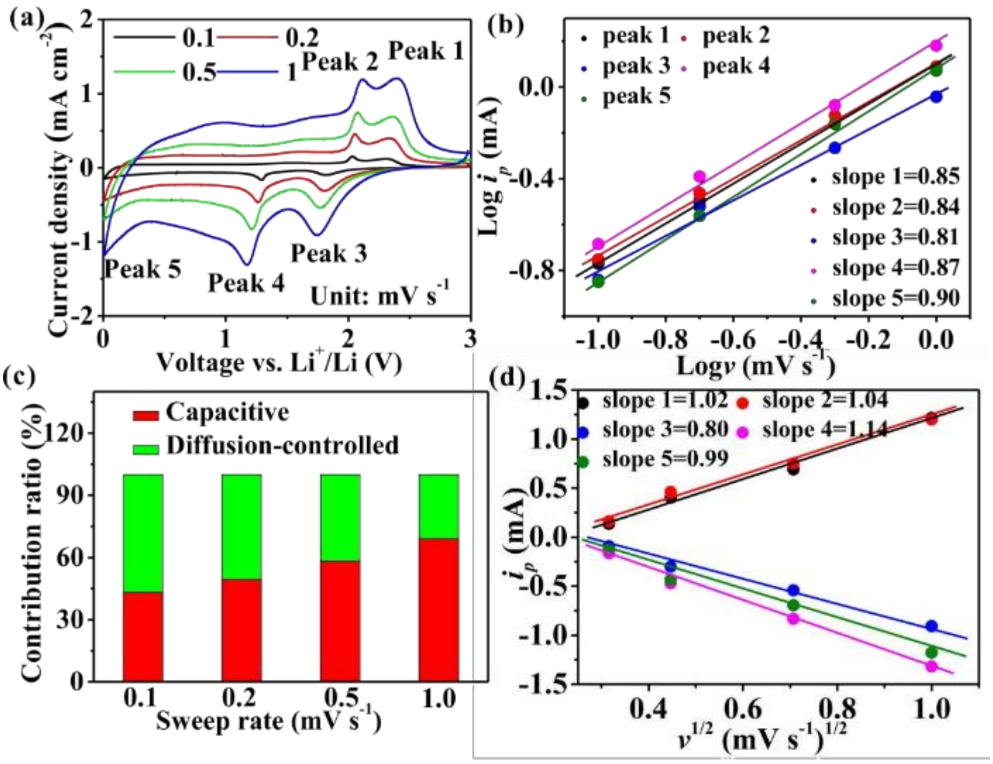

Fig. S21 (**a**) CV curves at different sweep rates, (**b**) Log i_p against Log v at peaks 1-5, (**c**) the percentages of pseudocapacitive contribution at different sweep rates, and (**d**) i_p versus $v^{1/2}$ at peaks 1-5 of $CoMoS_2$/C-III

Table S9 Comparison of $_{i+}$(cm^{2} s^{-1}) of the samples prepared in this work with the recently reported MoS_2-based LIB anode materials. The comparison value is the average value in Fig. 4f

Samples	D_{Li+}	Average D_{Li+}	References
$CoMoS_2$/C-II	7.05×10^{-10}-1.67×10^{-9}	1.19×10^{-9}	**This work**
MnS-MoS_2	10^{-14}-10^{-13}	$5x10^{-14}$	Adv. Funct. Mater. **31**, 2007132 (**2021**)
MoS_2	10^{-15}-10^{-14}	$5x10^{-15}$	Adv. Funct. Mater. **31**, 2007132 (**2021**)
MoS_2/C	$4.48x 10^{-18}$	$4.48x10^{-18}$	Chem. Eng. J. **372**, 665–672 (2019)
Mn-doped MoS_2/C	$2.51x10^{-16}$	$2.51x10^{-16}$	Chem. Eng. J. **372**, 665–672 (2019)
MoS_2-C	$1x10^{-15}$ -$1x10^{-9}$	$5x10^{-13}$	ACS Appl. Mater. Interfaces **8**, 22168-22174 (2016)
TiO_2/MoS_2	$3.12x10^{-14}$-$6.67x10^{-14}$	$4.9x10^{-14}$	J. Alloys Compounds **892**, 162075 (2021)

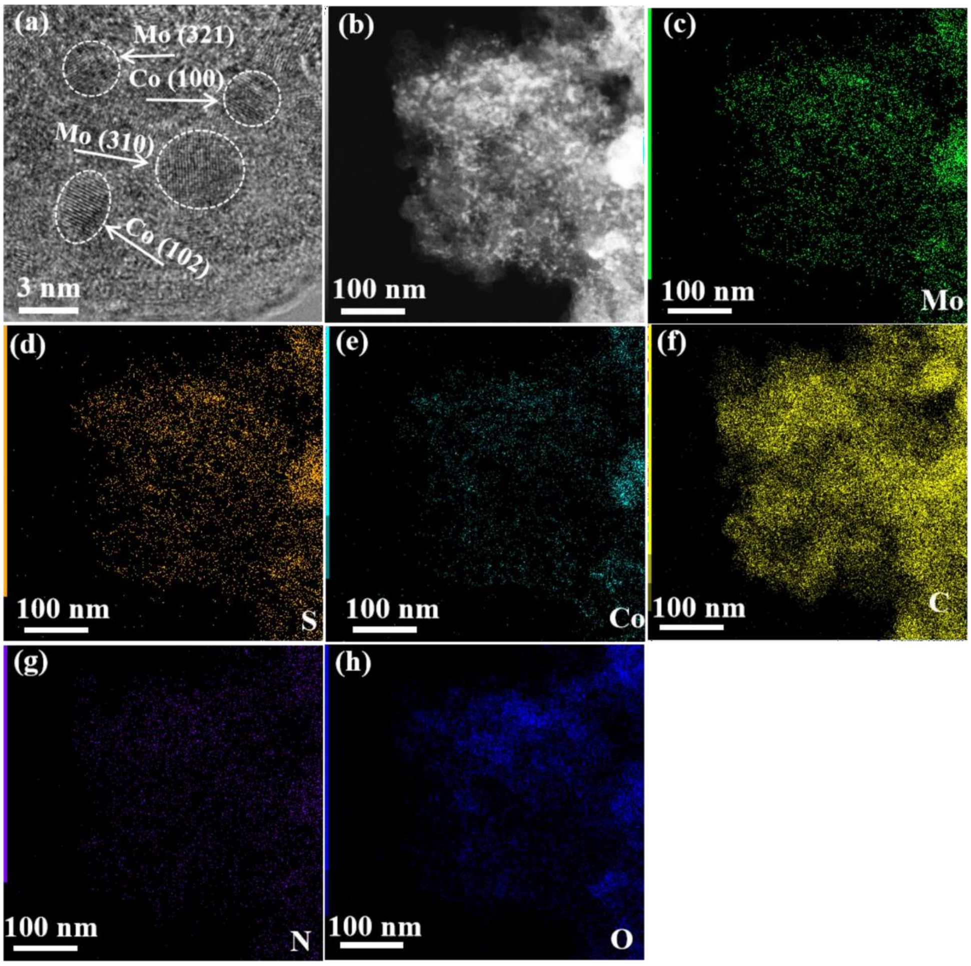

Fig. S22 (**a**) TEM image, (**b**) HAADF image of $CoMoS_2$/C-II electrodes after discharging to 0.01 V and corresponding EDS mapping of (**c**) Mo, (**d**) S, (**e**) Co, (**f**) C, (**g**) N, (**h**) O

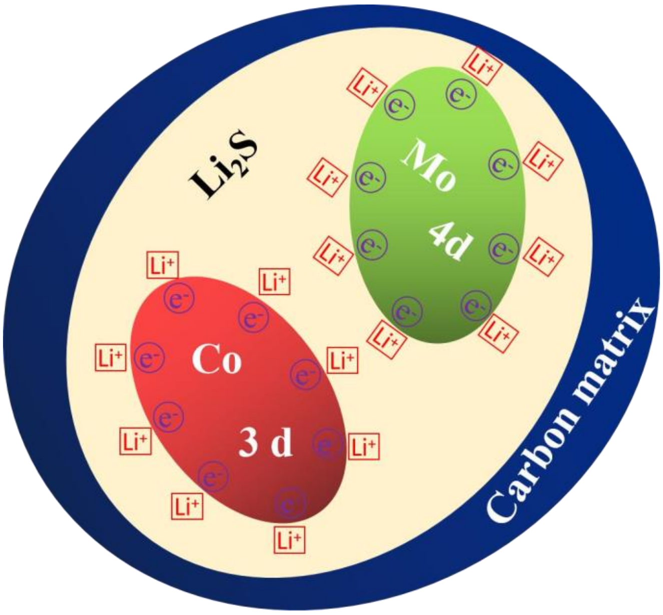

Fig. S23 The schematic diagram of the formation of space charge zone

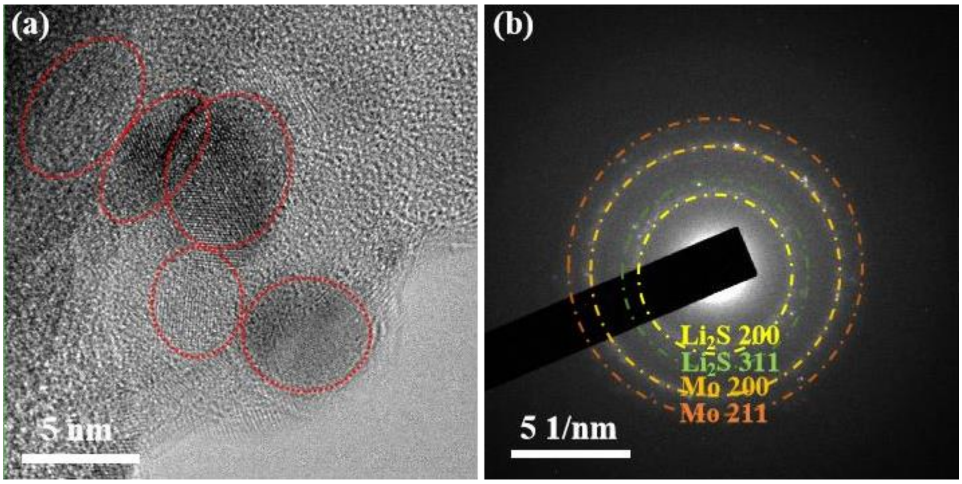

Fig. S24 *Ex-situ* TEM characterizations. (**a**) TEM images of MoS_2 electrode after discharging to 0.01 V, and (b) corresponding SAED pattern

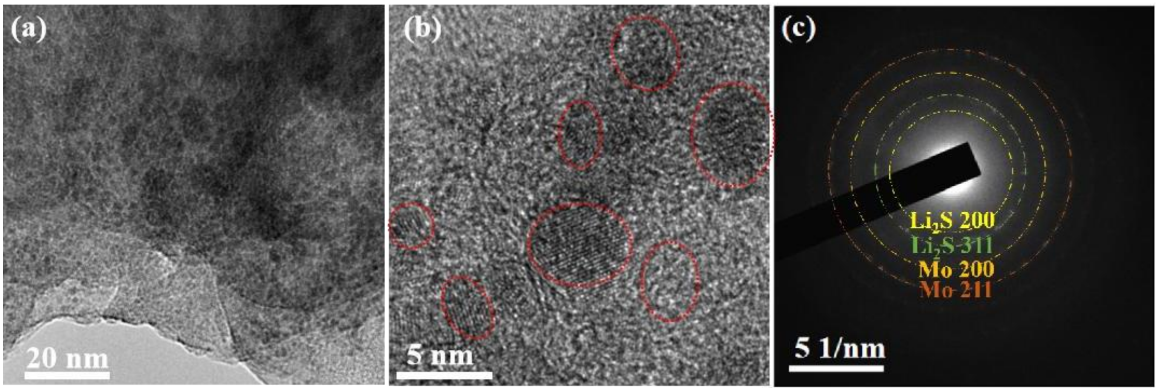

Fig. S25 *Ex-situ* TEM characterizations. (**a**, **b**) TEM images of MoS_2/C electrode after discharging to 0.01 V, and (**c**) corresponding SAED pattern

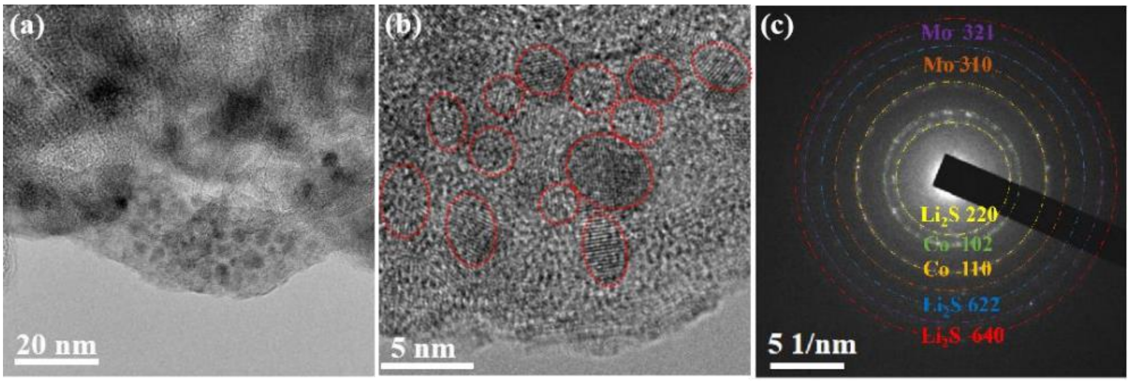

Fig. S26 (**a**, **b**) TEM images of $CoMoS_2$/C-I electrode after discharging to 0.01 V, and (**c**) corresponding SAED pattern

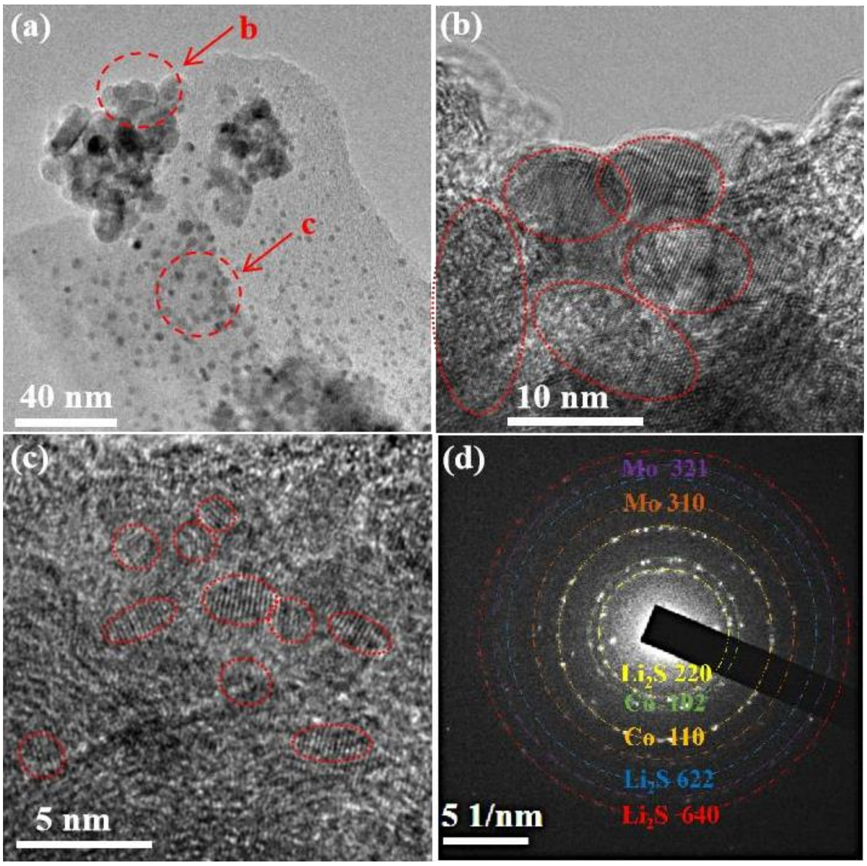

Fig. S27 (**a**, **b**) TEM images of $CoMoS_2$/C-III electrode after discharging to 0.01 V, and (**c**) corresponding SAED pattern. Image (**b**) is taken from red circle pointed by red arrow b in image (**a**). Image (**c**) is taken from red circle pointed by red arrow c in image (**a**)

Note: The larger nanoparticles are obtained in Fig. S27b, which should be ascribed to the conversion reaction of large-sized Co_3S_4. The smaller nanoparticles are obtained in Fig. S27c, which should be ascribed to the conversion reaction of Co-doped monolayer MoS_2. The average size of Mo and Co nanoparticles is calculated based on the amount of particles in Fig. S27a, and the size of particles in Figs. S27b, c, which is about 3.5 nm

Table S10 Electrochemical performances of MoS_2-based anode materials in LIBs full cell. C_C-charge capacity (mAh g^{-1}), C_R-capacity retention (%), M_L-mass loading (mg cm^{-2}), J-current density (A g^{-1}, based on cathode), N_C-cycle number, NA-not available.

Samples	C_R	M_L	J	N_C	References
**$CoMoS_2$/C-II**	**95.1**	**2.0**	**0.017**	**100**	**This work**
**$CoMoS_2$/C-II**	**90.2**	**2.0**	**0.17**	**200**	**This work**
**$CoMoS_2$/C-II**	**80.2**	**2.0**	**0.68**	**500**	**This work**
MnS-MoS_2	~ 73	1.2	0.5	350	Adv. Funct. Mater. **31**, 2007132 (2021)
SnS_2/MoS_2/CFC	63.5	1.5-2 mg cm^{-2}	0.15	50	Chem. Eng. J. **356**, 483-491 (2019):
C-MoS_2	80	~ 1.0	0.19	50	Chem. Eng. J. **428**, 131103 (2022)
N-GRs/MoS_2	86	1.0	0.1	200	Chem. Eng. J. **408**, 127269 (2021)
MoS_2-NT	~ 89	1.0	0.1	100	J. Alloys Comp. **907**, 164499 (2022)
MoO_2@MoS_2/rGO	81.6	1.5	0.2	40	Electrochim. Acta **364**, 136996 (2020)

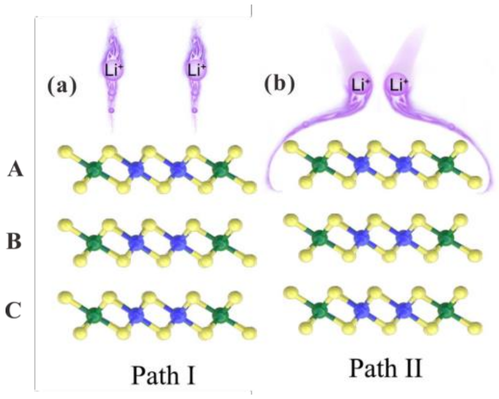

Fig. S28 Migration paths of Li^+ to from up to down. (**a**) Path I, cross the basal plane of layer A to transport; (**b**) Path II, bypass the basal plane of layer A to transport

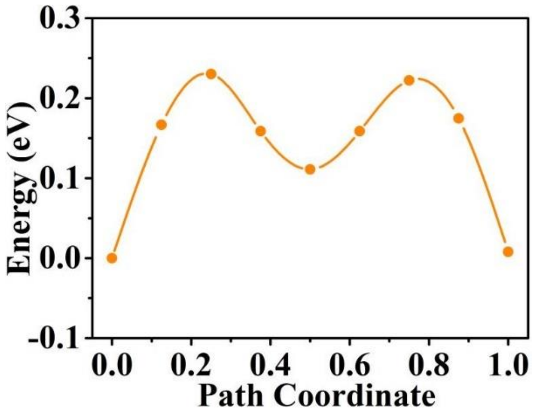

Fig. S29 Li^+ diffusion energy barrier in the configuration of migration paths in Fig. 7g in the manuscript
